# Improved eIF4E Binding Peptides by Phage Display Guided Design: Plasticity of Interacting Surfaces Yield Collective Effects

**DOI:** 10.1371/journal.pone.0047235

**Published:** 2012-10-19

**Authors:** Weizhuang Zhou, Soo T. Quah, Chandra S. Verma, Yun Liu, David P. Lane, Christopher J. Brown

**Affiliations:** 1 Bioinformatics Institute, Agency for Science, Technology and Research (A*STAR), Singapore; 2 p53 Laboratory, Agency for Science, Technology and Research (A*STAR), Singapore; 3 Department of Biological Sciences, National University of Singapore, Singapore; 4 School of Biological Sciences, Nanyang Technological University, Singapore; University of South Florida College of Medicine, United States of America

## Abstract

Eukaryotic initiation factor (eIF)4E is over-expressed in many types of cancer such as breast, head and neck, and lung. A consequence of increased levels of eIF4E is the preferential translation of pro-tumorigenic proteins (e.g. c-Myc and vascular endothelial growth factor) and as a result is regarded as a potential therapeutic target. In this work a novel phage display peptide has been isolated against eIF4E. From the phage sequence two amino acids were delineated which improved binding when substituted into the eIF4G1 sequence. Neither of these substitutions were involved in direct interactions with eIF4E and acted either via optimization of the helical capping motif or restricting the conformational flexibility of the peptide. In contrast, substitutions of the remaining phage derived amino acids into the eIF4G1 sequence disrupted binding of the peptide to eIF4E. Interestingly when some of these disruptive substitutions were combined with key mutations from the phage peptide, they lead to improved affinities. Atomistic computer simulations revealed that the phage and the eIF4G1 derivative peptide sequences differ subtly in their interaction sites on eIF4E. This raises the issue, especially in the context of planar interaction sites such as those exhibited by eIF4E, that given the intricate plasticity of protein surfaces, the construction of structure-activity relationships should account for the possibility of significant movement in the spatial positioning of the peptide binding interface, including significant librational motions of the peptide.

## Introduction

eIF4E initiates cap-dependent translation by binding to the cap structure (m^7^GTP) found at the 5′ end of mRNA. eIF4E is part of the large eIF4F complex which includes other proteins such as eIF4G and eIF4A. eIF4F forms a complex with the 40S ribosomal subunit and eIF3, which then shuttles along the 5′-untranslated region (5′-UTR) of the mRNA until it arrives at the AUG initiation codon. [1], [2] This is followed by complexation of the 40S subunit to the 60S ribosomal subunit, resulting in the 80S initiation complex, which is then ready to initiate the elongation cycle. Cap-dependent translation by eIF4E is regulated by the PI3K/Akt/mTOR pathway. [1] The interaction of eIF4E with eIF4G, as part of the eIF4F complex, is competitively blocked by the binding of the 4E-binding proteins (4E-BPs) to eIF4E. Hyperphosphorylation of the 4E-BPs by mTOR disrupts their interaction with eIF4E and allows eIF4E to recruit mRNA to the eIF4F complex. [3] mRNAs are also hypothesized to compete with one another for binding to the eIF4F complex and for delivery to the ribosomes. The short, unstructured 5′-UTRs of most cellular mRNAs enable the eIF4E containing complex to scan readily for the translation initiation codon (AUG) and allow optimal translation even when the active eIF4F complex is limiting. In comparison, the lengthy, G+C-rich, highly structured 5′-UTRs typical of proto-oncogenic mRNAs (e.g. cyclin D1, VEGF) hinder scanning by the initiation complex and recognition of the AUG start codon. This leads to the mRNAs being translated poorly, which is further attenuated when the active eIF4F complex is limiting. Although eIF4E regulates translation globally, it contributes to malignancy by enabling the increased translation of mRNAs with highly structured 5′UTRs when overexpressed. [4] This renders eIF4E a potential target for anti-cancer therapeutics. [5].

eIF4E can be specifically inhibited in several ways e.g. by kinase inhibitors of the mTOR complex [5], antisense RNA treatments that target eIF4E [6], cap analogues that attenuate mRNA binding [7] and peptidomimetics that mimic the eIF4G1 interaction with eIF4E. [8] The goal of peptidomimetics is to synthesize small molecule compounds that can mimic the spatial placement of amino acid side chains that occur at protein-protein interaction sites. [9] However this process is complex, time intensive, and does not guarantee the generation of a sub-micromolar compound. An alternative approach is to harness the potential of peptides as they are capable of achieving high affinities together with specificity. Hence the development of technologies that can enhance their cell permeability will enable the generation of novel tools and potential therapeutics e.g. the use of cell penetrating peptides (such as TAT and penetratin) [10], nanoparticles [11]–[12] and peptide stapling. [13], [14] In view of these developments in technologies that can guide the delivery of peptides to cells and target tissues, designing high affinity and selective peptides by a process of combinatorial design and rational engineering is necessary.

**Figure 1:**
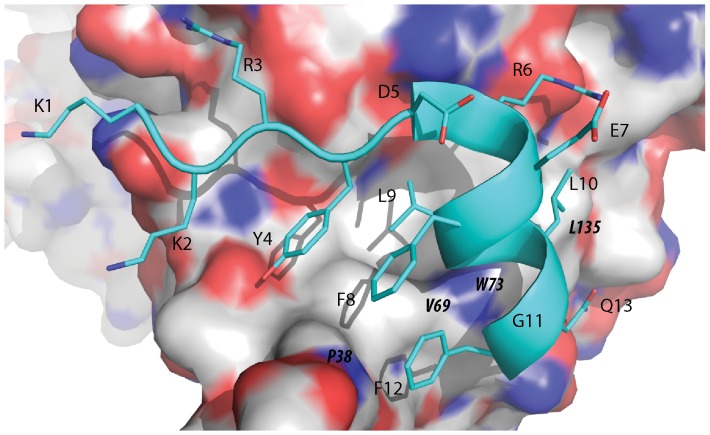
Structure of the eIF4G1 peptide in complex with eIF4E. The crystal structure of the eIF4G1 wild type peptide (cyan) in complex with eIF4E (2W97) showing key interactions between the peptide and the protein. Locations of eIF4E surface residues are shown in bold and italics on the protein surface representation, whilst eIF4G1 peptide residues are non-italicized.

Crystallographic studies have revealed that peptides derived from eIF4G1 and 4EBP1, which contain the critical residues responsible for their interactions with eIF4E, are molecular mimics of each other. [15] Both peptides when bound to eIF4E, as observed in the co-crystal structures, are approximately 50% α-helical but contain negligible helical content in solution. [15] Brown et al have demonstrated that the α-helix in the unbound eIF4G1 peptide could be stabilized by the incorporation of 2 non natural amino acids, 1-aminocyclopentanoic acid and c-α methyl L-phenylalanine, in aqueous solution. [16] The 4E-BP1 and eIF4GI peptides both posses a YXXXXLΦ motif (Φ signifies any hydrophobic residue). [15] The conserved tyrosine is located on an extended strand at the N-terminus of the α-helix of the bound peptides, whilst the two other conserved residues of the motif are found on the helix itself (see figure 1). The tyrosine makes multiple van der Waal contacts with eIF4E while its side chain hydroxyl forms a hydrogen bond with the carbonyl backbone of P38 of eIF4E. The conserved leucine exploits a shallow cavity on the surface of eIF4E. It also interacts with W73 of eIF4E via a hydrogen bond between its backbone and the indole nitrogen of the tryptophan. The conserved hydrophobic residue packs against V69 and L135 of eIF4E (see figure 1). In addition, D625 in the bound peptide is involved in a typical N-capping motif of the first turn of the helix, where its side chain engages in two stabilizing hydrogen bonds to the backbone amides of E627 and F628. [17].

In this study we isolated a novel phage derived peptide against eIF4E and identified two amino acid positions outside the conserved interaction motif that improved binding of the eIF4G1 sequence. Neither of these mutations were involved in direct interactions with eIF4E and acted either via stabilization of the helical capping motif in the bound complex or by restricting the conformational flexibility of the peptide. In addition, phage derived amino acid substitutions that had earlier been found to be disruptive for the binding of the eIF4G1 peptide, were discovered to be beneficial in the context of other substitutions that were introduced in the eIF4G1 peptide from the phage sequence, arising undoubtedly from the compensating influence of other specific amino acids. Understanding the nature of these substitutions in the phage and eIF4G1 peptide sequences has unveiled a striking relationship revealing how key amino acids influence the relationship between the precise location of the peptide binding site on eIF4E, stabilization of the bound peptide helix and the packing of the C-terminus of the peptide against eIF4E. The insights presented here should contribute to the design and evolution of peptidomimetics with higher affinity for eIF4E.

## Results and Discussion

### Delineation of Individual Amino Acids that Contribute to the Improvement of a Phage Selected and Native eIF4G1 Based Peptide

In our previous work [16] we had established how two amino acids that lie outside the conserved motif (YXXXXLΦ) improved the binding of a peptide to eIF4E by stabilizing its helical conformation. In order to see if this sequence could be improved further, a peptide (12mer) phage display library (NEB) was panned against biotinylated eIF4E. After three rounds of selection, a single phage peptide sequence was selected with the sequence ^1^SLHYSRDQLVAL^12^. The selection against eIF4E preserved the well known and well characterized interaction motif maintaining the Y at 624, L at 629 and the hydrophobic position at 630 seen in the eIF4G1 protein (corresponding to positions 4, 9 and 10 in the peptide numbering system used in the remainder of this article). However the rest of the sequence is intriguingly different from the corresponding eIF4G1 peptide sequence (^1^KKRYDREFLLGF^12^). Unfortunately the phage sequence (termed PHAGE) proved insoluble and highly intractable to work with, causing no observable shift in the thermal stability assay, so it was decided to change the N-terminal SLH sequence to KKR as seen in the original eIF4G1 peptide sequence.

Modification of the N-terminal of the PHAGE peptide generated a soluble peptide (termed PHAGESOL) that produced a shift of 9.83±0.1°C in the thermal stability assay which corresponds to an estimated K_d_ of 77 nM (see table 1). The equivalent eIF4G1 peptide (KKRYDREFLLGF) has an estimated K_d_ of 460 nM, which is approximately 6-fold weaker than the PHAGESOL peptide. In order to delineate the origins of the enhanced affinity of the PHAGESOL peptide over the eIF4G1 sequence, we synthesized a set of peptides introducing amino acids from PHAGESOL individually into the eIF4G1 sequence. The relevant sequences were ordered and ranked using the thermal stability assay. Interestingly there were two key substitutions that improved the affinity of the eIF4G1 sequence, a D to S substitution at position 5 and a G to A substitution at position 11. The D5S substitution caused an apparent 4.6-fold improvement in binding to eIF4E whilst the G11A mutation caused an ∼ 2.3-fold improvement (see table 1). Surprisingly two substitutions, L10V and F8Q disrupted binding to eIF4E by an order of magnitude (see table 1).

**Table 1:** Calculated K_d_s and derived ΔG° (Gibbs free energy of binding) for the interactions between eIF4E and the hybrid eIF4G1/PHAGESOL peptides.

Peptide	Sequence	Thermal Shift (T_m_, °C)	Estimated K_d_ derived from the T_m_ (nM)	SPR derived K_d_ (nM)		(ΔG°, cal mol^−1^)	
				K_eq_	K_kin_	K_eq_ derived	K_kin_ derived
**eIF4G1-WT**	^1^KKRYDREFLLGF^12^	7.03±0.1	460	580.2±16.7	523.9±61.6	−8500±20	−8550±70
							
**PHAGESOL**	^1^KKRYSRDQLVAL^12^	9.83±0.1	77	76.7±3.4	77.1±9.0	−9700±20	−9700±70
							
**eIF4G1-D5S**	^1^KKRYSREFLLGF^12^	8.97±0.07	100	103.0±2.3	99.9±6.2	−9520±10	−9540±40
**eIF4G1-G11A**	^1^KKRYDREFLLAF^12^	8.13±0.12	200	308.7±11.6	282.0±17.6	−8870±20	−8930±40
**eIF4G1-L10V**	^1^KKRYDREFLVGF^12^	3.9±0.03	4400	4537.0±621.7	2684.7±444.5	−7280±90	−7590±110
**eIF4G1-F12L**	^1^KKRYDREFLLGL^12^	6.87±0.12	520	761.4±63.8	718.1±98.0	−8340±50	−8370±90
**eIF4G1-F8Q**	^1^KKRYDREQLLGF^12^	5. 00±0.03	2000	1633.3±75.1	1777.0±406.1	−7890±30	−7840±150
**eIF4G1-E7D**	^1^KKRYDRDFLLGF^12^	6.87±0.18	520	784.4±21.5	758.8±70.7	−8320±20	−8340±60

The table shows the peptide sequences used to study the relevance of individual amino acid changes observed in the phage derived sequence. The peptides were characterized using a fluorescence based thermal denaturation method and by using SPR with eIF4E amine coupled to the chip surface. K_d_s were also derived from the respective thermal shift and SPR data. K_d_s were derived from the equilibrium responses (K_eq_) and from the association and dissociation phases (K_kin_) of the SPR data. The Gibbs free energy of binding (**ΔG°**) was calculated with the equation ΔG = −RT ln K_a_ using both dissociation constant values determined.

The peptides and their interactions with eIF4E were also examined with SPR (see table 1 and figure S1). Good agreement was observed between the K_d_s derived from the thermal stability data and the SPR data with the D5S and G11A substitutions confirmed as beneficial to eIF4E binding and the L10V and F8Q amino acid changes as significantly detrimental to binding. However, the binding of E7D and F12L mutant peptide to eIF4E, when measured using SPR, were much weaker than the eIF4G1 peptide, with K_d_s of 784.4±21.5 nM and 761.4±63.8 nM respectively (see table 1 and figure S1). These deleterious individual substitutions suggest that the higher affinity of PHAGESOL, which includes these substitutions, must arise from other compensatory changes. Interestingly, individual amino acid changes to the eIF4G1 peptide that are beneficial do not appear to interact directly with the eIF4E surface (see figure 1).

### Optimization of N-terminal Helical Capping Motifs in Short Peptides can Lead to Significant Improvements in the Interactions of eIF4E Binding Peptides

**Figure 2:**
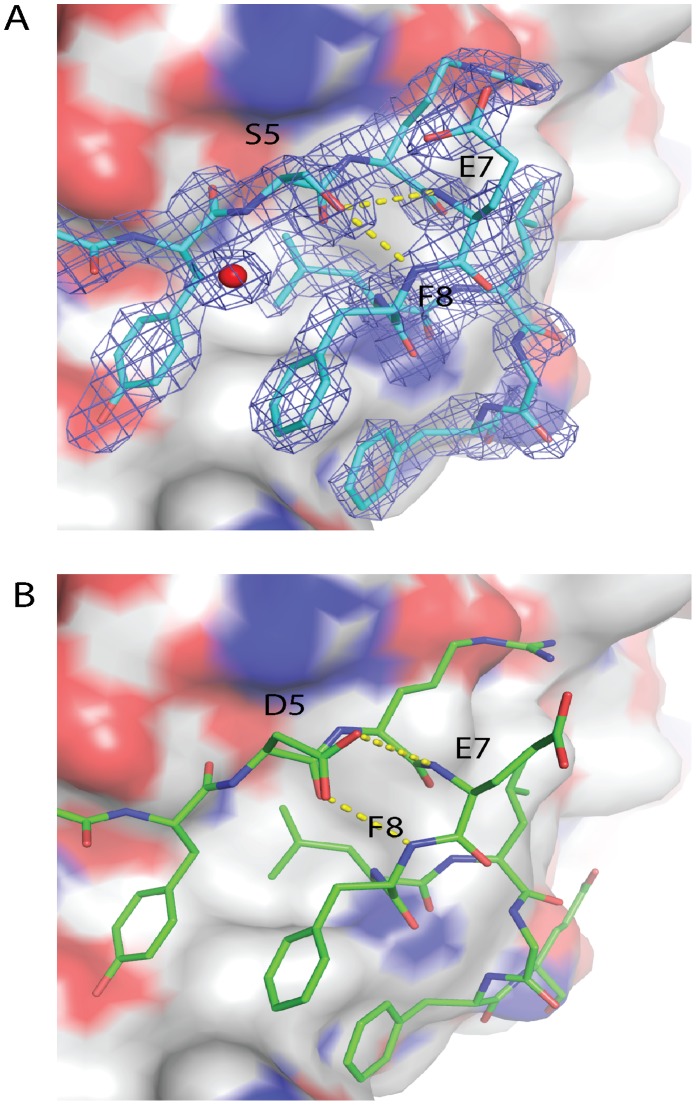
Structural comparison of N-Capping motifs either containing S or D at position 5 in eIF4E interacting peptides when bound to eIF4E. A) Crystal structure of the eIF4G1-D5S derivative peptide bound to eIF4E. It can be seen that S5 makes two optimized hydrogen bonds to the amide backbone groups of R6 and E7 located in the first turn of the helix of the bound peptide. The electron density for the eIF4G1-D5S peptide in the 2Fo-Fc map is shown with the blue mesh and is contoured at 1.5σ. **B)** D5 in the 2W97 structure bound to the eIF4G1 wild type peptide also makes these two hydrogen bonds but their geometry is not as optimal as that seen for the hydrogen bonds formed in the eIF4G1_D5S peptide.

eIF4E was successfully crystallized with the eIF4G1_D5S peptide which revealed the mechanism for the improvement in its affinity over the wild type peptide for this mutation. The hydroxyl group of the serine forms two optimal hydrogen bonds with the amide backbone groups of E7 and F8, which form the initial turn of the peptide α-helix seen in the crystal structure (see figure 2). The amide backbone groups in the first turn of the α-helix are not involved in hydrogen bonds with other residues in the helix and as a result are responsible for the positive dipole found at the N-terminal of helices. The positively charged N-termini of helices can be stabilized within proteins either by forming hydrogen bonds with a preceding residue outside the helix or with another residue from a discontinuous section of the protein. Residues identified to lie in the former category are Thr, Cys, and Asn and are referred to as the N-cap. It has been noted that among conserved and buried polar residues making hydrogen bonds to main chain NH functions in the N-terminal regions of α-helices, Cys has the highest propensity followed by Asp, His and Glu, whilst neutral residues like Ser, Thr and Asn have higher propensities when they are solvent accessible. [18].

The two hydrogen bonds formed by the hydroxyl group of the serine with the first turn of the helix are slightly shorter and hence energetically more favorable (3.2 and 3.4 Å respectively) when compared to the eIF4G1 peptide (3.3 and 3.5 Å respectively) in the crystal structures (see figure 2). In the alternative complexes in the asymmetric unit of both structures the bonds distances are 3.0 Å and 3.5 Å for the S5 modification compared to 3.5 Å and 4.4 Å for the D5 residue, respectively. To examine whether any other amino acid substitution could further stabilize the N-cap motif and improve binding, a new set of peptides was designed (see table 2 and figure S2). The amino acids chosen for insertion into the N-Cap position were Thr, Asn and Cys (see above). We also tested other amino acids based on their potential to form a hydrogen bond either with their side chains or resulting from the geometric constraints of their side-chains in comparison to Asp e.g. proline, glycine and glutamate. The S5 modified eIF4G1 sequence bound to eIF4E with a K_d_ of 103.0±2.3 nM, which is ∼ a 5.6 fold improvement over the original eIF4G1 sequence that contains a D. When the N-capping residue at position 5 was changed to a T no significant change was observed in the K_d_ compared to the eIF4G-D5S peptide (see table 2 and figure S2); this was not surprising since Ser and Thr have similar potentials to form hydrogen bonds with the first turn of the helix of the bound peptide.

**Table 2:** Calculated K_d_s and derived ΔG° (Gibbs free energy of binding) for the interactions between eIF4E and the N-Cap derivative peptides.

Peptide	Sequence	SPR derived K_d_ (nM)		(ΔG°, cal mol^−1^)	
		K_eq_	K_kin_	K_eq_ derived	K_kin_ derived
**eIF4G1**	^1^KKRYDREFLLGF^12^	580.2±16.7	523.9±61.6	−8500±20	−8550±70
**eIF4E-D5S**	^1^KKRYSREFLLGF^12^	103.0±2.3	99.9±6.2	−9520±10	−9540±40
**eIF4E-D5T**	^1^KKRYTREFLLGF^12^	110.2±1.8	104.9±7.2	−9480±10	−9510±40
**eIF4E-D5G**	^1^KKRYGREFLLGF^12^	467.8±28.1	439.5±26.0	−8630±40	−8660±40
**eIF4E-D5P**	^1^KKRYPREFLLGF^12^	683.7±13.6	717.3±31.2	−8400±10	−8370±100
**eIF4E-D5N**	^1^KKRYNREFLLGF^12^	444.5±1.6	388.9±1.6	−8660±10	−8740±10
**eIF4E-D5E**	^1^KKRYEREFLLGF^12^	749.0±57.7	692.4±31.2	−8350±50	−8390±30
**eIF4E-D5C**	^1^KKRYCREFLLGF^12^	436.5±6.4	458.0±19.8	−8670±10	−8640±30

The table shows the peptide sequences used to study the influence of alternative residues and their effects in capping the first turn of the α-helix when bound to eIF4E. K_d_s were determined using SPR with eIF4E immobilized on the chip surface. K_d_s were derived from the equilibrium responses (K_eq_) and from the association and dissociation phases (K_kin_) of the SPR data. The Gibbs free energy of binding (**ΔG°**) was calculated with the equation ΔG = –RT ln K_a_ using both dissociation constant values determined.

**Figure 3:**
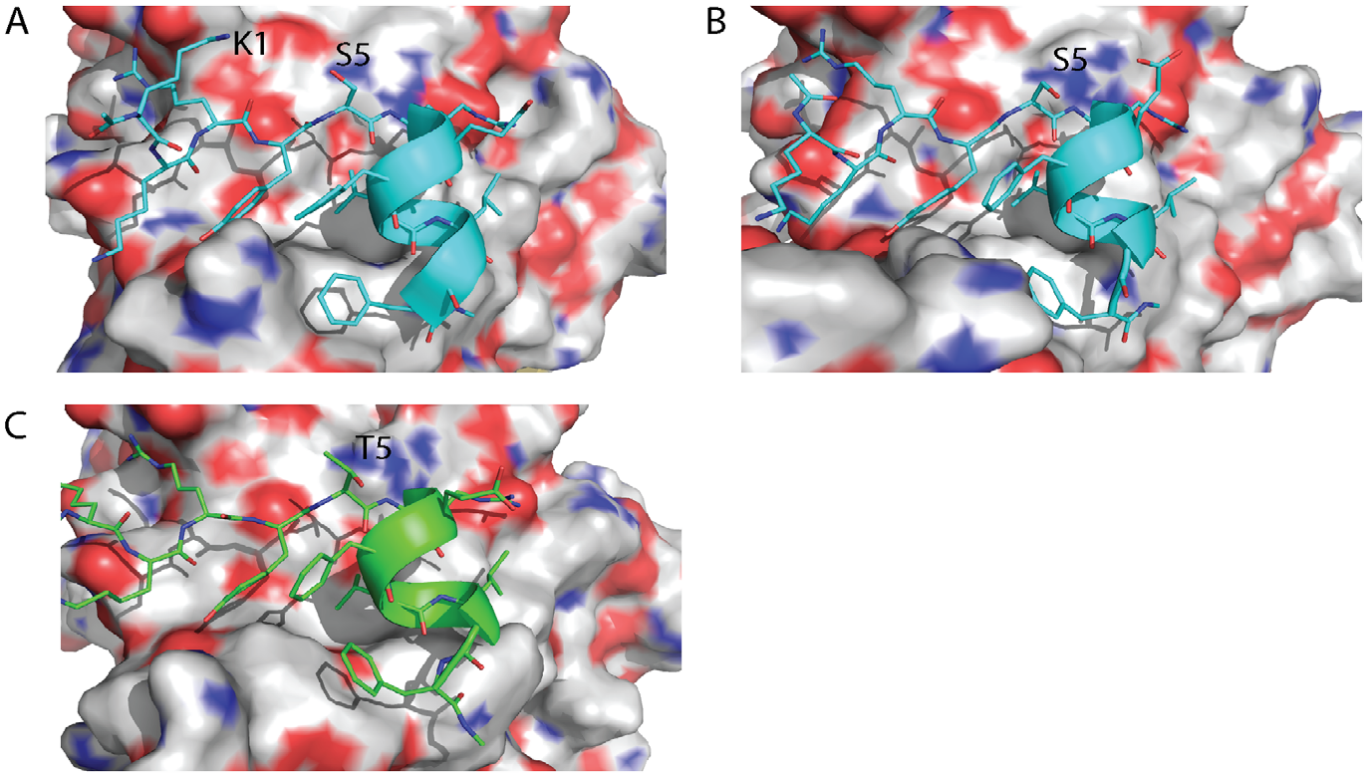
Comparison of the structural dynamics of N-Capping motifs, in peptides bound to eIF4E, containing either S or T at position 5. The S5 and T5 N-Capping residues show distinctly different behaviors with respect to each other in terms of the frequency of rotation of their side chains in their respective simulations when bound to eIF4E. **A)** The S5 side chain can form an interaction with the K1 side chain of the bound peptide and point away from the helix leaving the amides solvated. **B)** The S5 side chain can also form hydrogen bond interactions with the free amide groups of the first turn of the peptide’s helix. **C)** The T5 side chain can also make these interactions, however if the hydroxyl of the T5 interacts with K1, its methyl group will disrupt solvation of the free amide groups. Thus it is energetically more favourable for T5 to align its hydroxyl group towards the helix whist S5, which lacks the methyl, has more freedom to rotate, and engages in one of the two hydrogen bonds. Deviations in the planarity of the tyrosine and phenylalanine ring systems are within the tolerances of the torsional restraints of the MD simulations. [31].

Despite the similarities in binding energies, the S5 and T5 N-Capping mutations show distinct differences in the dynamics of their side chains when analyzed in 50 ns molecular dynamics simulations. The S5 side chain is able to sample several more rotameric states than the T5 side chain. The hydroxyl group of the T5 side chain spends a significant proportion of the simulation aligning itself towards the N-terminus of the peptide. The S5 side chain either forms an interaction with K1, leaving the amide groups of the helical turn solvated, or forms hydrogen bond interactions with the amide groups of the first helical turn. The T5 side chain can also perform these interactions, however if the hydroxyl of the T5 interacts with K1, its methyl group will disrupt solvation of the free amide groups. Thus it is energetically more favourable for T5 to align its hydroxyl group towards the helix while S5, which lacks the methyl, has more freedom to rotate, and engages in one of the two hydrogen bonds (see figure 3).

**Figure 4:**
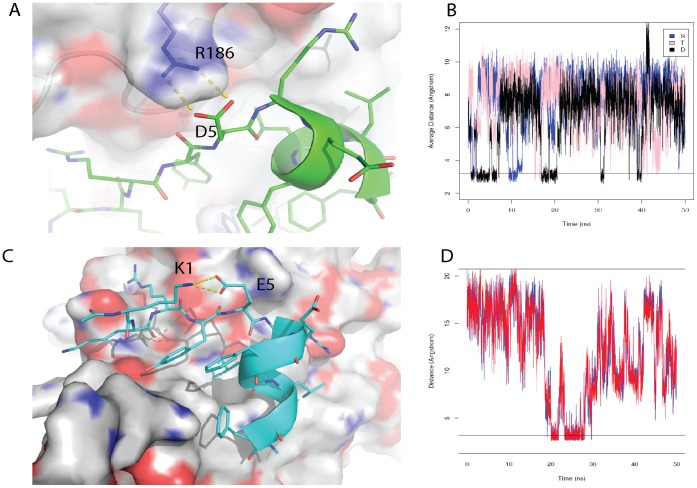
Detrimental interactions that attenuate peptide binding to eIF4E when D or E are incorporated at position 5. A) Snapshot from the 50 ns computer simulation of the eIF4G1 wild-type peptide bound to eIF4E. In the eIF4G1 wild type peptide the D5 side chain is able to hydrogen bond with R186 of the receptor, deforming the N-terminal helix of the bound peptide. This deformation causes E7 of the peptide to interact more frequently with R6, which is also found on the peptide. This interaction disrupts the electrostatic interaction between R196 found on the surface of eIF4E and E7 of the eIF4G1 peptide, which helps to stabilize the formation of the peptide:protein complex. **B)** The average distance between E7 and R6 throughout the 50 ns simulations for the bound peptides eIF4G1-D5S, eIF4G1-T5S and the wild-type peptide. The average distance frequently dips below 3.2 Å for the wild type peptide compared to the S5 or T5 derivative peptides, which indicates that E7 and R6 interact more frequently with each other and destabilize complex formation in the wild type peptide compared to the derivative peptides. **C)** A snapshot from the 50 ns simulation of the complex between eIF4G1-D5E and eIF4E, showing the formation of a loop-like structure preceding the N-terminus of the peptide. The formation of the loop structure arises from the electrostatic interaction of E5 of the peptide with K2 at the N-terminus of the peptide. **D)** The distances between the two O atoms (the red and blue lines on the plot) of the E5 side chain and the N atom of the K1 side chain were plotted over the course of the simulation. The plot reveals that for a significant portion of the simulation these residues are within 3.2 Å of each other indicating the formation of a stable electrostatic interaction. This interaction hinders the interaction of K1 with E132 on the surface of eIF4E and leads to further destabilization of the eIF4E-peptide complex. Deviations in the planarity of the tyrosine and phenylalanine ring systems are within the tolerances of the torsional restraints of the MD simulations. [31].

In the simulation of the eIF4G1 wild-type peptide, the D5 side chain occasionally forms hydrogen bonds with the R186 side chain of eIF4E, causing a slight deformation at the N-terminal end of the α-helix in the bound peptide (see figure 4). This deformation of the helix causes the side chain of R6, located in the peptide, to form intramolecular hydrogen bonds more frequently with E7 of the bound peptide. This reduces the hydrogen bonds between R6 and E132 of eIF4E, yielding a reduced interaction of the peptide with eIF4E. When the capping residue D5 is changed to S5 in the eIF4G1_D5S peptide, the deformation appears to be attenuated. This results in greater stabilization of E132 of eIF4E with R6 of the peptide (see Figure 4A and 4B), leading to improved interactions of the peptide with eIF4E.

When either G, C or N are incorporated at position 5, the K_d_ decreases slightly in comparison to the eIF4G1 WT peptide with K_d_s of 467.8±28.1 nM, 436.5±6.4 nM and 444.5±1.6 nM respectively (see table 2 and figure S2). The improvement in the affinity of these peptides lies in the absence of D5 and hence its interactions with R186 of eIF4E, which now allows for improved interactions between R6 and the surface of eIF4E. However, the G5, C5 and N5 mutant peptides have K_d_s that are approximately 4-fold weaker than the S5/T5 mutants. The side chain of C5 is less polar and correspondingly makes weaker hydrogen bonds, whilst G5 does not possess any hydrogen bond forming side chain and can only potentially make one through its backbone carbonyl. Additionally, the sulphur atom in C5 is larger than the oxygen atom in S5 and this provides steric hindrance in its movement; the C5 side chain behaves more akin to T5. When N5 is present at the N-Cap position, simulations suggest that it mainly interacts with the backbone amides of R6 and R7, located in the first turn of the helix, through its highly polar side chain. The reason for the higher dissociation constant compared to the S5 and T5 may be due to the fact that in the complexed state, N5 is constrained together with the rest of the peptide while in its uncomplexed state, the N will be largely solvated. Complexation will thus lead to entropic and desolvation penalties that will be higher than for S or T.

eIF4G1-D5E has the lowest affinity for eIF4E, with a K_d_ of 749±57.7 nM (see table 2 and figure S2). This increase in K_d_ is partially due to the longer side chain of E5 which sterically favors the g+ rotamer. This results in less energetically favorable hydrogen bonds with the free amide groups on the first turn of the α-helix. Additionally, simulations show that the E5 side chain preferentially interacts with R186 of eIF4E, with a lifetime occupancy of up to 80%. Interestingly, in contrast to the extended strand structure at the N-terminal of the bound peptide, E5 occasionally adopts a loop-like local structure (see figure 4D). This loop results from an intramolecular interaction between E5 and K2 of the peptide and prevents K2 from forming a favourable electrostatic interaction with D148 on the surface of eIF4E (which is characteristic of all the other peptides). The cumulative effects of these interactions likely lead to the observed lowered affinity.

The eIF4G1-D5P peptide binds eIF4E with a marginally improved K_d_ of 683.7±13.6 nM compared to that of eIF4G-D5E (see table 2 and figure S2). Here, all the amides of the first helical turn remain solvated but the φ angle of the P residue is restrained to approximately −75°. This restriction will have additional effects on the conformation of the extended strand structure, which contains the critical Y4 residue, and will probably affect the ideal geometry of interactions that Y4 makes with eIF4E. Hydrogen bond analysis of the simulation shows the existence of a hydrogen bond between the backbone atoms of P625 and L629 throughout the simulation that stabilizes the helix of the peptide, despite the P5 residue possessing no hydrogen bond forming side chain to interact with the first turn of the α-helix.

### Amino Acid Changes to the C-terminal of eIF4E Interacting Peptides Modulate their Affinity

**Figure 5:**
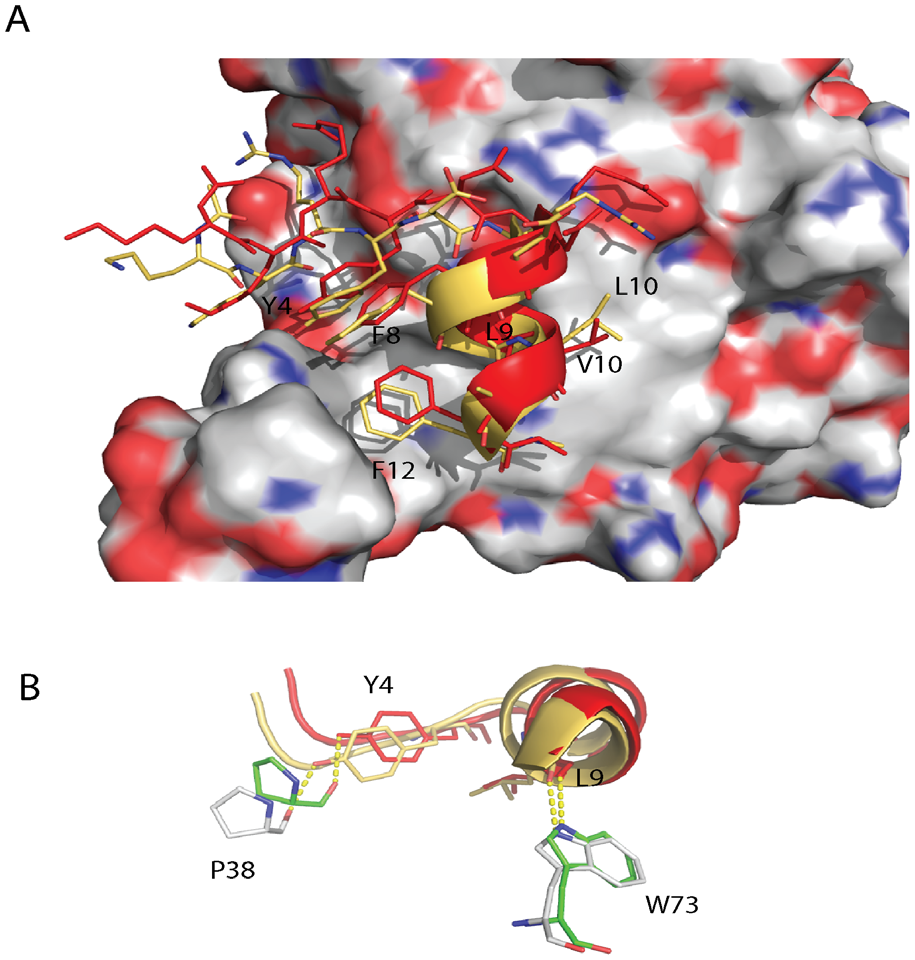
Amino acid changes to the C-terminal of eIF4E interacting peptides modulate their interactions with eIF4E. An overlay of two respective snapshots from the 50 ns simulations of eIF4G1-G11A (yellow) and eIF4G1-L10V (red). The G11A mutation restrains the C-terminal conformation of the peptide allowing it to pack more efficiently and reduce the entropic cost of binding. Interestingly in the eIF4G1-L10V peptide the mutation causes the peptide to shift its orientation along the planar surface of eIF4E. The change in orientation is essentially caused by V10 trying to pack at the position vacated by the L residue. V10 is unable to pack optimally as this would result in severe disruption of the packing interaction of F8 and F12 with the surface of eIF4E. However even the suboptimal packing of V10 leads to disruption of interactions made by the F8 and F12 side chains with the result that the F12 packs differently against eIF4E and F8 is displaced. The disruption of multiple interactions and the loss of the packing of L12 against L135 and W73 significantly attenuates the interaction of the eIF4G1-L10V peptide with eIF4E. **B)** Two key hydrogen bonds are involved in the interface between the conserved interaction motif of the eIF4E binding peptides and the protein itself. These hydrogen bonds exist between Y4 and L9 of the conserved motif and the eIF4E surface residues P38 and W73, respectively. The hydrogen bond between Y4 and P38 is significantly displaced spatially between the eIF4E:eIF4G1-G11A (peptide in yellow, eIF4E residues in white) and eIF4E:eIF4G1-L10V (peptide in red, eIF4E residues in green)) interfaces, but the geometries of the interactions between the complexes are maintained due to the flexibility of the N-terminal tail which P38 is located on. The hydrogen bond between the carbonyl of L9 and the side chain of W73 in contrast is reasonably invariant. Deviations in the planarity of the tyrosine and phenylalanine ring systems are within the tolerances of the torsional restraints of the MD simulations. [31].

The replacement of L10 in the eIF4G1 WT peptide by V10 severely attenuates binding activity whilst replacement of G11 with A11 leads to an improvement in the affinity (see table 1 and figure S1). The L10V and G11A substitutions were further studied with computer simulations over 50 ns and were either shown to disrupt or improve packing interactions against eIF4E via structural effects at the C-terminal end of the peptide. The L10V substitution abolishes the close packing seen for L10 against W74 and L135 of eIF4E. However V10 moves closer to the surface of eIF4E to occupy as much of the volume previously occupied by L10 (see Figure 5). This lateral movement of V10 causes conformational changes in the rest of the bound peptide with F12 moving in towards F8, which in turn rotates away from the surface of eIF4E. These conformational changes which involve the loss of the L10 packing interactions and changes in the manner in which F8 and F12 pack against eIF4E have a detrimental effect, with the K_d_ at 4537.0±621.7 nM.

The effect of these changes causes the helix of the peptide to move laterally across the surface of the protein (see figure 5) and to principally pivot around the conserved hydrogen bond made by the backbone carbonyl of L9 to the side-chain of W73. Interestingly the geometries of the two key hydrogen bonds formed between the conserved residues, Y4 and L9 of the two peptides with P38 and W73 of eIF4E respectively, are maintained. The Y4 hydrogen bond with P38 is significantly displaced between the two peptide protein complexes in figure 5. Only the intrinsic mobility of the flexible N-terminal tail of eIF4E, which P38 is located on, allows the geometry of the hydrogen bond to be retained in both complexes. In comparison the second hydrogen bond between L9 and W73 shows little variation emphasizing how the eIF4G1-L10V peptide pivots around this residue.

The G11A substitution causes a restriction in the φ/ψ angle distribution at this position, which provides an entropic reduction in the cost of binding by restraining the F12 residue into an optimal packing position against eIF4E (see figure 5). Additionally the G11A peptide causes no loss of packing interactions against the surface of eIF4E as seen for L10V. The stabilization in the packing arrangements of the G11A peptide results in an increase in the affinity of the peptide with K_d_ at 308.7±11.6 nM.

### Comparison of 4EBP1 and eIF4G1 Peptides Suggests that a Slightly Distorted Helical Structure is Possible for Packing against the Surface of eIF4E

**Figure 6:**
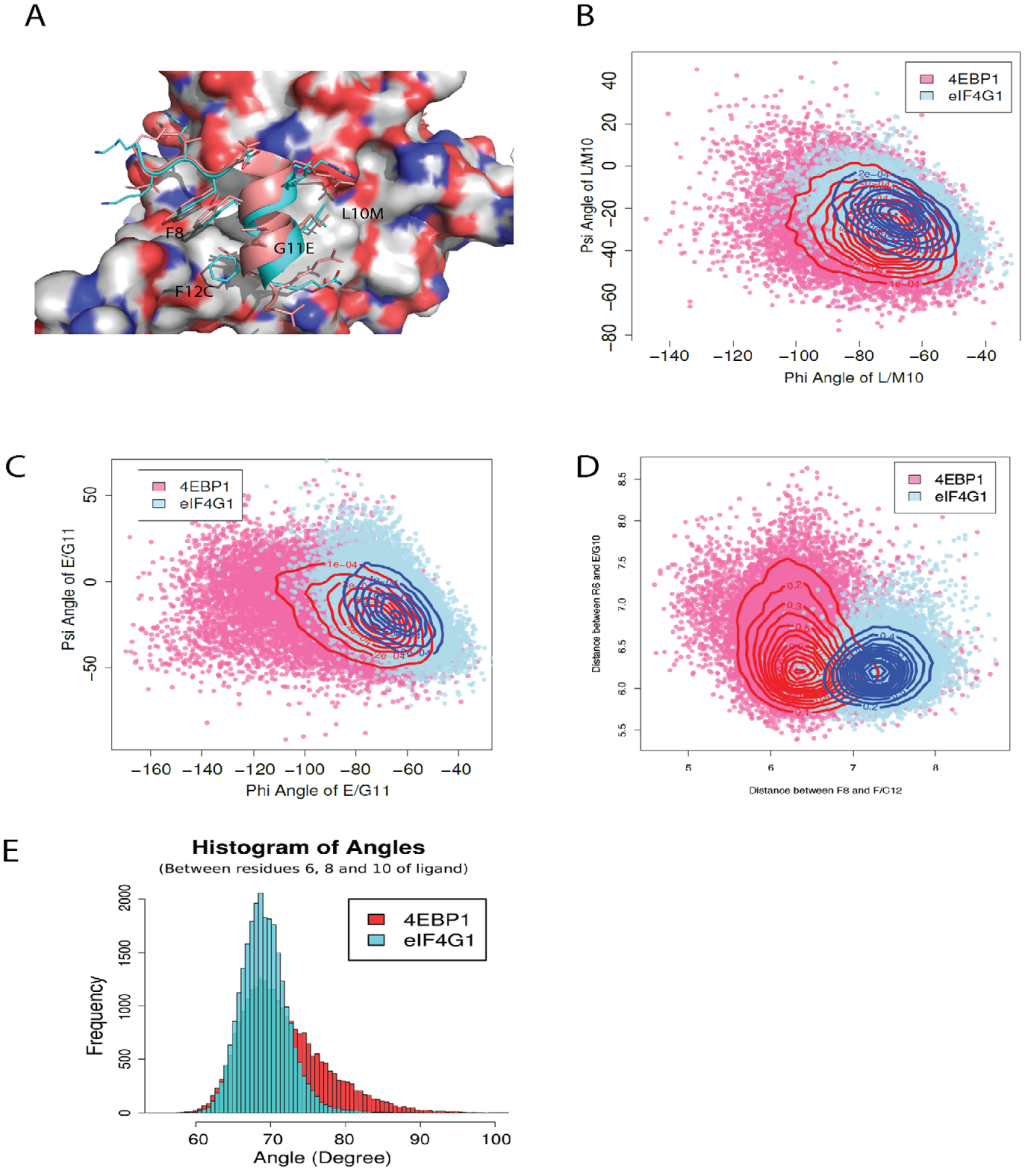
Comparison of 4EBP1 and eIF4G1 peptides suggests that eIF4E interacting peptides can form an ensemble of conformations when in complex with eIF4E. **A)** An overlay of two eIF4E crystal structures complexed with either a 4EBP1 (1EJ4) or eIF4G1 (2W97) derived peptide demonstrating the deviation in their C-terminal structural conformations. 4EBP1 is shown in salmon and eIF4G1 in cyan. **B)** A plot of the φ and ψ angle distribution, derived from the 50 ns simulations of the peptides eIF4G1 and 4EBP bound to eIF4E, for the residues L10 and M10 respectively. **C)** A plot of the φ and ψ angle distributions, derived from the 50 ns simulations of peptides eIF4G1 and 4EBP bound to eIF4E, for the residues G11 and E11 respectively. **D)** A plot showing the distribution of distances, for the peptides eIF4G1 and 4E-BP1 when bound to eIF4E, between the Cα atoms of residues 6 and 10 versus the distance between the Cα atoms of residues 8 and 12. The distances were calculated from their respective 50 ns simulations for both peptides. **E)** A histogram of the angular distribution between the Cα atoms of positions 6, 8 and 10 of the eIF4G1 and 4EBP1 peptides from the 50ns simulations respectively.

If the crystal structure of the eIF4G1 peptide in complex with eIF4E is overlaid with the eIF4E:4EBP1 complex (4EBP1 is an eIF4E inhibitory protein that competes with eIF4G1 for binding to eIF4E), it is immediately apparent that the axis of the helix in the eIF4G1 peptide deviates with respect to the helical axis of the 4EBP1 peptide (see figure 6) from position 10 onwards. The structural differences between these peptides were further examined with 50 ns molecular dynamics simulations of the 4EBP1 and eIF4G1 peptides in complex with eIF4E. The distribution of the φ and ψ angles around position 11 in both peptides during the simulations was examined revealing that the range of φ angles were similar for G11 and E11 (see figure 6). However, the average φ angle, which affects the C_α_-C_α_ distance, is approximately 10° smaller in magnitude for eIF4G1 than it is for 4EBP1. Also the spread of φ/ψ values sampled by G11 is considerably less diffuse than for E11 (see figure 6). When the same analysis is carried out for position 10 in both peptides, i.e. L10 in eIF4G1 and M10 in 4EBP1, the φ/ψ angles are very similar (see figure 6B) but the distribution around position 10 in the eIF4G1 peptide is more constrained. These results indicate that the structural differences between the eIF4G1 peptide and the 4EBP1 peptide at the extreme C-terminus result in larger sampling of the conformational space by the 4EBP1 peptide. To further investigate the structural differences between the C-termini of the two peptides, the C_α_-C_α_ distances between amino acid positions 6 and 10 and between positions 8 and 12 were measured. The C_α_–C_α_ distance between residues 6 and 10 remains relatively unchanged for both cases throughout the simulations whilst there is a clear change in the distribution of distances for residues 8 and 12 between both peptides (see figure 6). These differences confirm that F12 in eIF4G1 deviates away from the α-helical conformation observed in 4EBP1. If the angle between the C_α_ atoms of amino acids 8, 10 and 12 is calculated for both peptides, the eIF4G1 peptide possesses a more constrained distribution whereas 4EBP1 shows a heavy-tailed distribution. The deviation of the eIF4G1 helix appears to result from the packing between the F residues at positions 8 and 12 against each other and against eIF4E. In addition, the presence of G at position 11 allows the helix to flex (see figure 1) in order to accommodate the optimal packing of F8 and F12 against eIF4E. This deviation in the α-helical structure of the two peptides suggests that the mutations cumulatively and collectively influence the dynamics of the peptide and modulate its interactions with eIF4E.

### Phage Display Panning Gives Rise to Compensatory Amino Acid Changes via Hydrophobic Packing Effects and Cumulative Stabilization of the Bound Helix

To further investigate the mechanics underlying the peptide interactions of eIF4E with the phage derivative peptide and the eIF4G1 peptide, the amino acids that were shown to be beneficial or detrimental to binding, were removed from either sequence and replaced with the corresponding residue from the other peptide. In PHAGESOL, the detrimental V10 was mutated to the naturally occurring L found in the eIF4G1 WT peptide and in the case of the eIF4G1 WT peptide, D5 was changed to the optimal N-capping residue S and G11 to the conformationally less mobile A11 from PHAGESOL. The exchange of V10 for L in the PHAGESOL V10L peptide produced an approximate two-fold improvement in binding, with a K_d_ of 37.2±1.6 nM whilst the changes in the eIF4G1 WT peptide produced a peptide (termed eIF4G1-OPT) with a slightly weaker K_d_ of 52.2±1.4 nM (see table 3 and figure S3). This clearly indicates that the difference in affinities between these two peptides (PHAGESOL V10L and eIF4G1-OPT) lies in the unchanged residues. The inclusion of V at position 10 in the phage sequence by the panning process is intriguing and is clearly more detrimental to binding in the wild type eIF4G1 sequence than in the phage derived sequence. To investigate this differing detrimental effect of the V10 residue in different peptide sequences we investigated the relationship of positions 8 and 12 with position 10.

**Table 3:** Calculated K_d_s and derived ΔG° (Gibbs free energy of binding) for the interactions between eIF4E and the derivative peptides used to study the relationship of amino acids at positions 8 and 12 in relation to the presence of the V residue present at position 10.

Peptide	Sequence	K_d_ from SPR (nM)		(ΔG°, cal mol^−1^)	
		K_eq_	K_kin_	K_eq_ derived	K_kin_ derived
PHAGESOL	^1^KKRYSRDQLVAL^12^	76.7±3.4	77.1±9.0	−9700±20	−9700±70
eIF4G1-OPT	^1^KKRYSREFLLAF^12^	52.2±1.4	59.4±2.9	−9920±20	−9850±30
PHAGESOL-Q8F	^1^KKRYSRDFLVAL^12^	195.6±6.3	183.8±15.4	−9140±20	−9180±50
PHAGESOL-L12F	^1^KKRYSRDQLVAF^12^	111.5±2.7	120.0±5.3	−9470±10	−9430±30
PHAGESOL-Q8F/L12F	^1^KKRYSRDFLVAF^12^	348.4±20.9	373.5±33.1	−8800±40	−8760±50
VL-PHAGESOL-Q8F	^1^KKRYSRDFLLAL^12^	49.6±4.3	47.15±1.5	−9950±50	−9980±20
VL-PHAGESOL-Q8F/L12F	^1^KKRYSRDFLLAF^12^	69.94±6.4	62.0±3.5	−9750±60	−9820±30
VL-PHAGESOL	^1^KKRYSRDQLLAL^12^	37.2±1.6	34.7±1.2	−10,120±30	−10,170±20
VL-PHAGESOL-L12F	^1^KKRYSRDQLLAF^12^	43.21±0.34	40.7±3.8	−10,040±10	−10,070±60
eIF4G1-QL	^1^KKRYDREQLLGL^12^	1093.0±49.9	1087.7±106.1	−8120±30	−8130±60
PHAGESOL-D7E	^1^KKRYSREQLVAL^12^	51.6±1.8	52.9±2.1	−9930±10	−9920±20

The table shows the peptide sequences used to study the relationship of amino acids at positions 8 and 12 in relation to the presence of the V residue present at position 10. K_d_s were determined using SPR with eIF4E immobilized via amine coupling on the chip surface. K_d_s were derived from the equilibrium responses (K_eq_) and from the association and dissociation phases (K_kin_) of the SPR data. The Gibbs free energy of binding (**ΔG°**) was calculated with the equation ΔG = -RT ln K_a_ using both dissociation constant values determined.

The Q8F and L12F mutations in the original PHAGESOL peptide resulted in peptides with up to 2.4-fold increase in K_d_ (see table 3 and figure S3, peptides PHAGESOL-Q8F and PHAGESOL-L12F respectively). If these two amino acid changes are combined (peptide PHAGESOL-Q8F/L12F) a more dramatic change is observed with the K_d_ increasing 4.5-fold. However if V10 is mutated to L (as in the wild-type eIF4G1 peptide) in all three peptides, (see peptides VL-PHAGESOL-Q8F, VL-PHAGESOL-Q8F/L12F, VL-PHAGESOL and VL-PHAGESOL-L12F in table 3 and figure S3) the detrimental effect of the F substitutions are attenuated significantly. The cumulative effect of these substitutions on the affinities of this group of peptides suggests allosteric effects.

**Figure 7:**
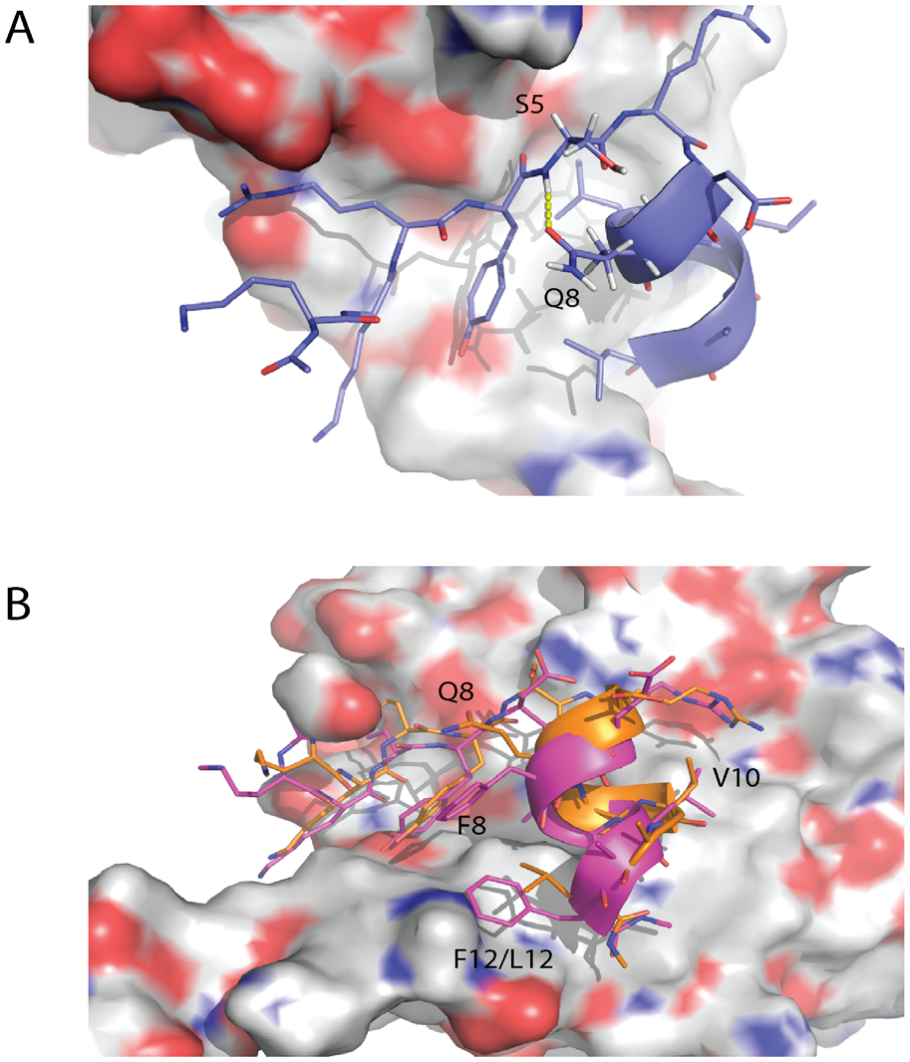
Q8 stabilizes the N-terminal end of the α-helix in eIF4E interacting peptides and facilitates the librational movement of the PHAGESOL peptide across the surface of eIF4E. A) A representative snapshot showing the formation of an intra-molecular hydrogen bond between the side chain of Q8 and the backbone amide of S5. The formation of this hydrogen bond stabilizes the N-terminal portion of the helix in the bound peptide. The snapshot was taken from the simulation for the KKRYSRDQLLAL peptide bound to eIF4E. B) Overlay of representative snapshots from the PHAGESOL (KKRYSRDQLVAL) and eIF4G1-G11A (KKRYDREFLLAF) peptide simulations when bound to eIF4E. V10 of the PHAGESOL (orange) peptide orients itself and the helical turn it is located on, into a position where it can occupy the volume of space that would otherwise be occupied by the conserved L as shown here on the eIF4G1 derivative peptide (magenta). The movement of V10 across the surface of eIF4E results in the helical segment of the peptide spatially re-orientating itself in contrast to the other derivative peptides. This conformational change is facilitated by the presence of Q8 that stabilizes the N-terminal helix of the peptide and forms few interactions with eIF4E in contrast to F8 in eIF4G1-G11A. Deviations in the planarity of the tyrosine and phenylalanine ring systems are within the tolerances of the torsional restraints of the MD simulations. [31].

Simulations show that V10 in the PHAGESOL (KKRYSRDQLVAL) peptide packs closely against the surface of eIF4E, in a manner reminiscent of the eIF4G1_L10V peptide, but much more efficiently (see figure 7). The conformation of the Val residue as well as the helical turn it is located on enables it to occupy the volume of space that would otherwise be occupied by L10 in the wild-type peptide. The librational motion of V10 across the surface of eIF4E results in the helical segment of the peptide spatially re-orientating itself in contrast to the other derivative peptides (see figure 7). In the eIF4G1 peptide such a conformational change would severely disrupt the packing of the F8 and F12 side chains against eIF4E, which in turn would prevent V10 from packing optimally against L135 and W74 of eIF4E (as is seen for the eIF4G1-L10V peptide). However in the PHAGESOL sequence, F8 is replaced by Q8, which forms less packing interactions against the surface of eIF4E, but instead forms an intramolecular hydrogen bond via its side chain to the backbone amide of S5. The net effect of this change is to stabilize the N-terminal of the helix and allow it to move collectively. This allows the peptide to orient itself and pack more optimally against eIF4E. Further, the F12L change in the PHAGESOL peptide results in a side-chain that can pack more efficiently against eIF4E, which enhances the peptide’s new orientation and ensures optimal packing of V10 (see figure 7).

**Figure 8:**
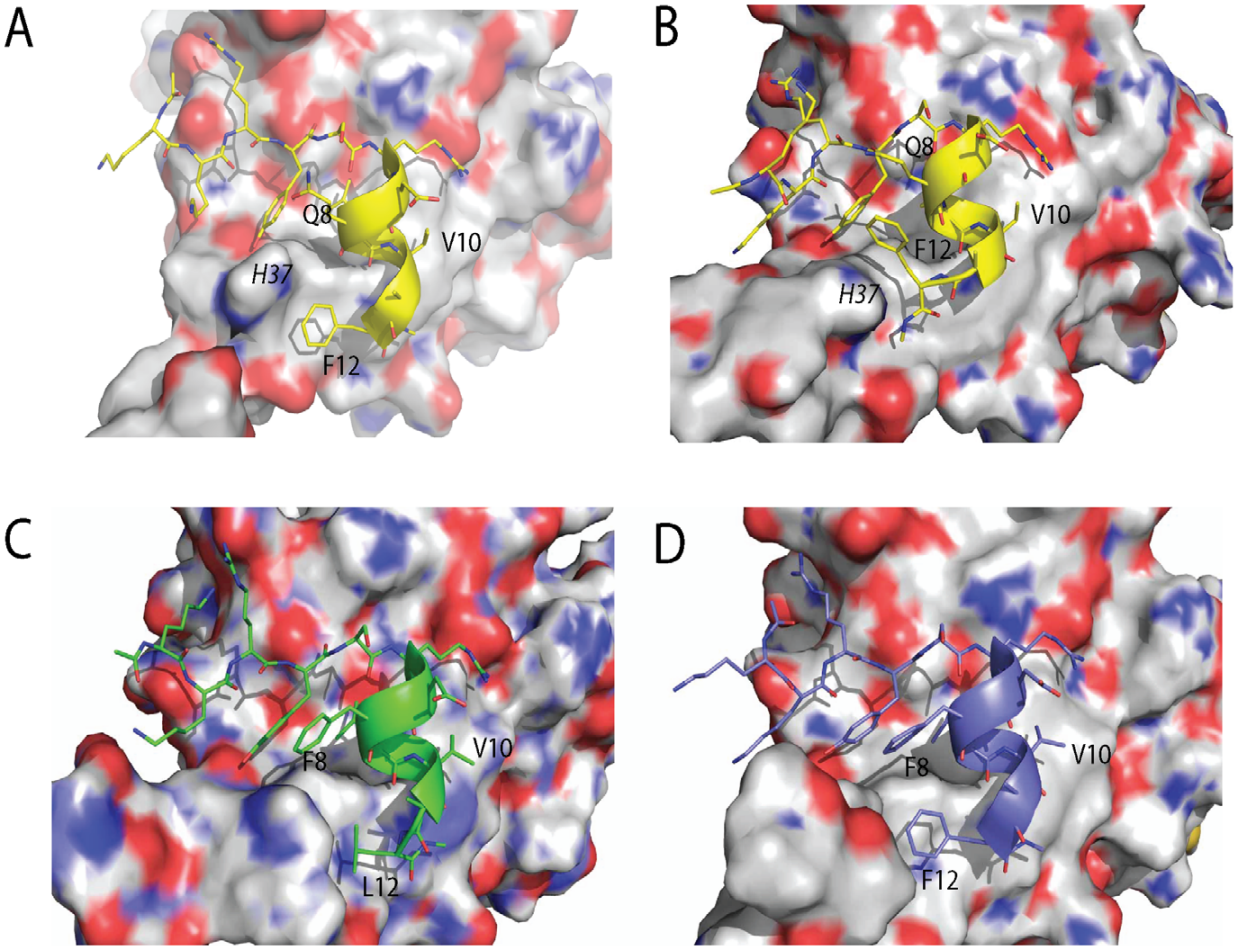
The presence of F8 impedes the structural fluctuations observed at the C-terminal of eIF4E interacting peptides. When L12 is replaced by F12 to generate the PHAGESOL-L12F peptide, two contrasting interactions are formed with eIF4E. **A)** In the first interaction H37 of eIF4E can intercalate itself between Q8 and F12 of the peptide in the volume of space usually occupied by F8 in other derivative peptides. **B)** The second conformation sees the unwinding of the helical turn at the C-terminus of the peptide allowing F12 to partially occupy the space next to Q8 previously occupied by H37. H37 instead now forms a stacking interaction with F12. It is the result of these alternative packing arrangements that prevents V10 from traversing into the optimal packing position seen in the PHAGESOL peptide against W73 and L135. In the two derivative PHAGESOL-Q8F and Q8F/L12F peptides (**C** and **D** respectively) with F located at position 8 there is minimal change in conformation observed between the two simulations and both have poor affinities for eIF4E. F8 forms favorable interactions with the surface of eIF4E and prevents any significant movement of the helix across the surface of the protein. In **C)** and **D)** both L12 (PHAGESOL-Q8F) and F12 (PHAGESOL- Q8F/L12F) pack against eIF4E with L12 making the more optimal interactions as reflected by the higher affinity of this peptide. Deviations in the planarity of the tyrosine and phenylalanine ring systems are within the tolerances of the torsional restraints of the MD simulations. [31].

When L12 is replaced by F12, the peptide can adopt two very different conformations against eIF4E. In the first conformation, H37 of eIF4E can intercalate itself between the side chains of Q8 and F12 of the peptide, in the space usually occupied by F8 in other derivative peptides e.g. PHAGESOL-Q8F, PHAGESOL-Q8F/L12F (see Figure 8). The second conformation sees the unwinding of the helical turn at the C-terminus of the peptide allowing F12 to partially occupy the space next to Q8 previously occupied by H37 (see figure 8). The H37 sidechain now forms a stacking interaction with the sidechain of F12. It is the result of these alternative packing arrangements that prevents the V10 residue from traversing into the optimal packing position seen in the PHAGESOL peptide (where it packed against W74 and L135). However the packing interactions made by F12 fail to alleviate the loss of the L10V interactions. In the two derivative PHAGESOL peptides, which contain the Q8F mutation, there is minimal change in conformation observed between the two simulations and both have poor affinities for eIF4E. F8 forms favorable interactions with the surface of eIF4E and prevents any significant movement of the helix across the surface of the protein to optimize the packing of V10. L12 most likely makes more complementary hydrophobic interactions with the surface residues than F at this position resulting in a slightly more potent peptide. However both peptides fail to compensate for the interactions lost by the L10V mutation.

**Figure 9:**
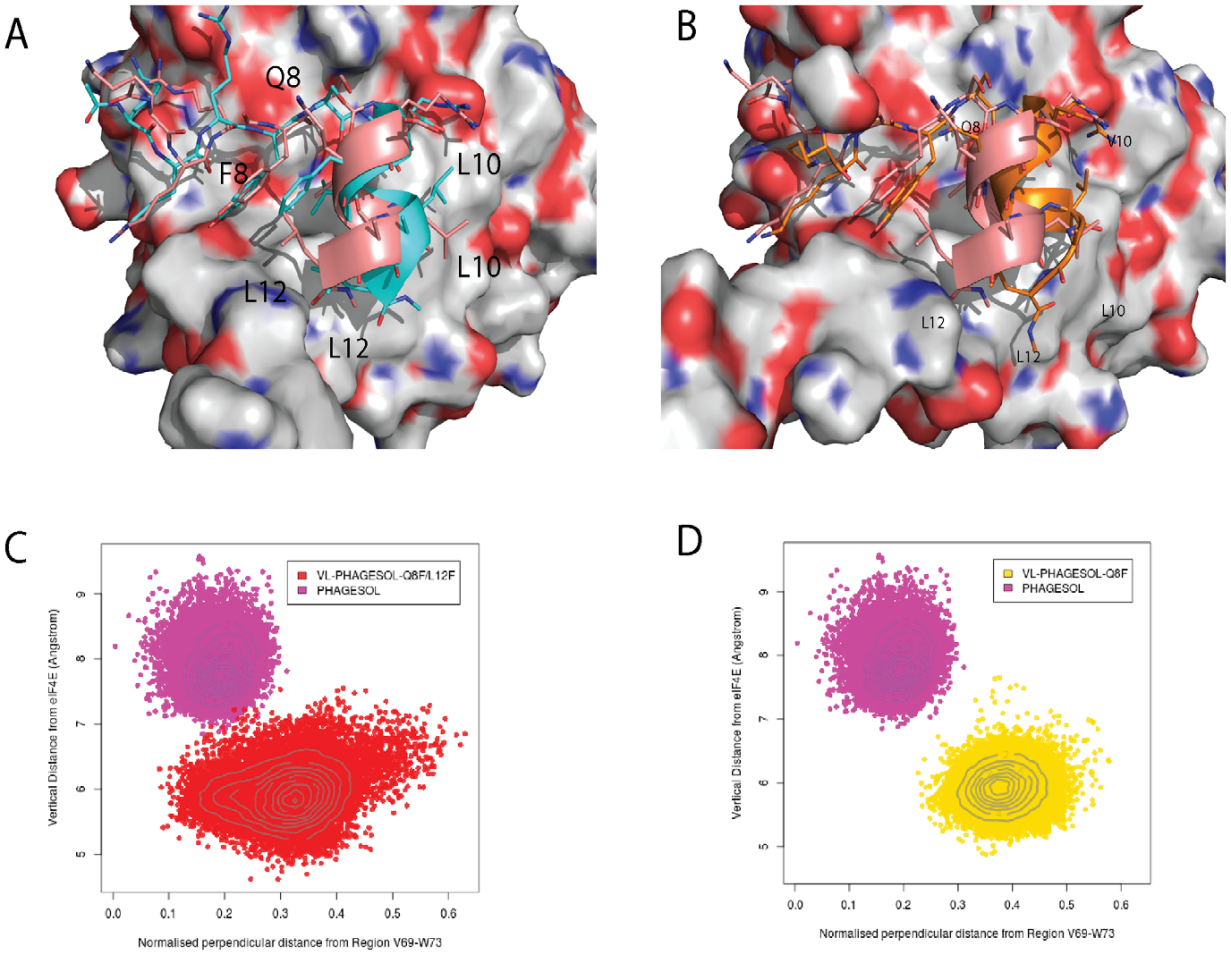
The precise sites of interaction for eIF4E interacting peptides are non-identical and display distinctive conformational differences. **A)** Overlay of representative snapshots from the VL-PHAGESOL-Q8F (cyan) and VL-PHAGESOL (salmon) peptide simulations bound to eIF4E. The deviation in helical movement between these two peptides is dramatic with F8 in the VL-PHAGESOL-Q8F peptide forming extensive interactions with eIF4E and preventing the lateral movement across the surface of eIF4E seen for VL-PHAGESOL. The lateral movement of VL-PHAGESOL is facilitated by Q8, which forms a hydrogen bond with S5 of the bound peptide, and also forms less interactions with eIF4E. It also allows the C-terminal of the helix to unwind and allow L12 to pack optimally into the volume that F8 would occupy in the other peptide. **B)** Overlay of representative snapshots from the PHAGESOL and VL-PHAGESOL peptide simulations bound to eIF4E. These peptides only differ at the position 10 with PHAGESOL containing a V and VL-PHAGESOL possessing an L. The presence of Q8 allows the helix to move more independently enabling the residue at position to dictate the final packing arrangements of the peptide. V10 packs optimally in the PHAGESOL peptide against the surface of eIF4E which induces the lateral movement of the peptide. L12 can still pack optimally. L10 in the VL-PHAGESOL peptide also packs against a hydrophobic area of eIF4E, closely located to where V10 packs, but due to the greater length of the alkyl chain allows the peptide to pack in a different conformation with eIF4E. A conformation where the C-terminal end unwinds to allow L12 to pack into an alternative optimal position against eIF4E. **C)** and **D)** are plots showing the distribution of the relative positions of the helical portion of the eIF4E bound peptide with respect to the surface of the protein throughout their individual simulations. The plots show the distinct conformational differences of the peptides in their interactions with eIF4E. The PHAGESOL peptide (magenta) and VL-PHAGESOL (yellow) both have very distinct conformational populations whilst the VL-PHAGESOL-Q8F/L12F peptide (red) has a much more dispersed population that overlaps with the conformational space of the VL-PHAGESOL peptide (see figure 4S for definition of the conformational measurement made). Deviations in the planarity of the tyrosine and phenylalanine ring systems are within the tolerances of the torsional restraints of the MD simulations. [31].

When V10 is replaced with L in the VL-PHAGESOL derivative peptide, they all have improved affinities for eIF4E compared to the PHAGESOL peptides (see table 3 and figure S3). The affinities for this set of peptides are much closer together in terms of magnitude than the V10 variant PHAGESOL peptides. When an F is present at position 8 (VL-PHAGESOL peptides Q8F and Q8F/L12F) the residue forms similar interactions to those observed in the V10 variant peptides and prevents the helix of the peptide changing its position on the surface of eIF4E (see figure 9). In contrast to the V10 variant peptides L10 is still able to form favorable interactions with W74 and L135 of eIF4E due to its longer alkyl chain length and this explains the increased affinity of these peptides for eIF4E. The C-terminal L12 or F12 residues both pack in the most optimal packing positions available on the surface of eIF4E with the L12 modified peptide possessing a marginally more potent K_d_ (see table 3 and figure S3). The VL-PHAGESOL and VL-PHAGESOL-L12F peptides both contain Q at position 8 and have even lower K_d_s against eIF4E (see table 3 and figure S3). The Q8 residue again forms an intra-molecular hydrogen bond against the amide backbone of S5 and makes fewer interactions with eIF4E in contrast to F8, which forms extensive hydrophobic interactions with the protein surface. The result of the Q8 substitution is to allow the helix of the peptide to re-orient itself. With the L10 substitution present the helix is less likely to transverse in the direction of L135 of eIF4E as the side chain of L10 is much longer than the side chain of V10. Thus the peptide can easily satisfy the packing requirements of L10, which in turn allows the helix to exploit more optimal packing positions depending on the substitution present at the C-terminus of the peptide. In the case of the VL-PHAGESOL peptide the C-terminal can unwind slightly, which together with the re-oriented helix, allows the L12 side chain to pack favorably into the volume of space that was occupied by F8 in the VL-PHAGESOL-Q8F peptide. The VL-PHAGESOL-Q8F peptide also possesses an L residue at position 12 but the interactions of F8 prevent it from making the same interactions (see figure 9). To further validate the observations made from the individual trajectories of the simulations, distribution plots of the relative positions of the helical portion of the PHAGEOL and VL-PHAGESOL variant peptides in relation to the surface of eIF4E were derived (see figure 9C and 9D) from their respective simulations. These plots confirmed the distinct conformational differences in the interactions being formed between eIF4E and the two peptides. The other individual peptide variants were also examined and showed similar differences to the PHAGESOL peptide (see figure S4).

Intriguingly, if Q8 and L12 are simultaneously substituted into the eIF4G1 peptide (eIF4G1_F8Q/F12L, see table 3 and figure S3), the K_d_ increases to ∼1000nM (∼2-fold greater than the eIF4G1 peptide). The Q8 and L12 mutations individually also attenuate the interactions between the eIF4G1 peptide and eIF4E (see table 1) with the replacement of F8 with Q8 having a drastic effect. This result emphasizes the importance of the conformationally restrictive A residue at position 11 and the presence of S5 as the N-Cap. The A11 residue plays an important role in stabilizing the C-terminal conformation of the peptide and orientating the less bulky L residue at position 12 into a favourable position to interact with eIF4E. However S5 stabilizes the first helical turn of the bound peptide and can form an additional interaction with the side chain of glutamine when it is present at position 8. This interaction further stabilizes the N-terminus of the bound helix. However when Q replaces F8 in the eIF4G1 sequence (see Table 1) this helix stabilizing interaction cannot form with the N-capping residue, as it is more bulky and carries a strong charge, which disrupts the intramolecular hydrogen bond. Thus the Q8 substitution has no beneficial effect in the context of the wild type sequence and the favourable interactions of F8 with the surface of eIF4E are lost, which results in a peptide (eIF4G-F/Q) with a K_d_ 3-fold weaker than that of the wild type peptide.

### Conclusion

α-helices are commonly found at the interfaces of interacting proteins and it is a common objective of most peptidomimetic design strategies to either mimic them with small molecules or stable short linear peptides. A major objective is to stabilize these short peptides into helical motifs prior to binding, to minimize the entropic costs associated with the helical bound conformations; this has been achieved by techniques such as stapling. [14] Here we show that in addition to stabilizing the helical conformation prior to the binding of a peptide to a protein, gains in potency can be achieved by further stabilization of the bound form of the peptide by optimizing intramolecular hydrogen bonds that in turn lead to enhanced packing interactions with the target surface.

SPR measurements and atomistic simulations of eIF4E and its complexes with a variety of peptides reveal how the conserved motif (YXXXXLΦ) in the peptides is responsible for binding eIF4E. The Y4 residue, as revealed by crystallography, makes a critical hydrogen bond between its hydroxyl group and the carbonyl backbone of P38 of eIF4E. However the F8 residue anchors the peptide to a precise location on eIF4E, as it makes extensive hydrophobic interactions, preventing the peptide from librating extensively on the surface of the protein (e.g. PHAGESOL peptides Q8F and Q8F/L12F as well as peptides VL-PHAGESOL Q8F and Q8F/L12F). When this F is replaced by Q, improvements in K_d_ are dependent on the presence of S at position 5 (instead of D5 present in the eIF4G1 sequence). S5 principally stabilizes the first turn of the bound α-helix via 2 hydrogen bonds to the backbone amides of E7 and F8. In contrast to F8, Q8 has a side chain that can form an intramolecular hydrogen bond with the S5 backbone amide, whilst making less extensive interactions with eIF4E, which allows the peptide more latitude in how it interacts with eIF4E. The S5 and Q8 mutations together stabilize the N-terminal of the bound helix, whilst decreasing hydrophobic interactions with the surface of the protein at position 8, thus allowing the peptide to find an alternative packing arrangement on the protein surface to more optimally satisfy the other peptide side chains (see peptides PHAGESOL and VL-PHAGESOL).

The movement of the peptide along the surface of eIF4E is essentially determined by the presence of a Q at position 8 and the size of the hydrophobic residue at position 10 (YXXXXLΦ), which is part of the recognition motif for eIF4E. The hydrophobic residue at position 10 forms a key interaction with a clearly demarcated hydrophobic patch on the surface of eIF4E, which is constituted principally by L135 and W73. When a V is located at position 10 and there are no anchoring interactions on the far side of the peptide (e.g. if F8 has been replaced by Q8) the peptide will transverse across the protein’s surface in order to optimise the hydrophobic interactions made by the Val (see figure 7). If position 10 is occupied by either a Leu or a Met, this movement across the protein surface is no longer seen. This originates in the longer alkyl chains of L and M side chains, which allows the hydrophobic interactions with L135 and W73 to be satisfied without the need for significant transverse movement of the helix. The packing of substitutions at position 12 depend on the substitutions present at positions 8 and 10. For example in the presence of Q8 and V10, as in the PHAGESOL-L12F peptide, F12 forms two alternative packing arrangements involving the H37 residue of eIF4E; however if Phe is present at position 8 the interactions formed by the amino acid at position 12 are very different (see figure 8).

Another interesting aspect of these design changes is whether increases in affinity are accompanied by changes to specificity. By optimizing interactions that stabilize the bound peptide, specificity in addition to affinity can be gained as they are only formed upon recognition of the correct surface. However, we observe that the precise interactions sites of the derivative peptides against eIF4E do not overlay exactly (see figure 9), and are principally determined by the underlying surfaces which are plastic (as is seen in the mobility of H37 of eIF4E) and by the librational movement of the peptide as determined by the residues present at positions 8 and 10. For example PHAGESOL and VL-PHAGESOL, which only differ at position 10 with the replacement of a Val by a Leu, bind optimally onto surfaces that differ from each other (see figure 9). This highlights the difficulty of designing peptides and the need to include the flexibility of the interacting surfaces and their mutual modulation in the design process. This strengthens the importance of computer simulations which can be a powerful tool to reveal the dynamics of such surfaces and the manner in which they can modulate each other. The diversity observed here further reveals the existence of rugged binding landscape that characterizes this interaction hotpsot on eIF4E where several energy minima exist close to each other. The discovery of librational motions of the peptides on this fluctuating surface of eIF4E is a new dimension that adds to our previous discovery of a dynamically flexing peptide on the fluctuating surface of MDM2. Indeed, the plasticity of H37 of eIF4E (see figure 8) adds a new layer of intricacy to the rational design process. [19].

The current study outlines the importance of combining atomistic simulations, structural characterizations and careful SPR K_d_ determinations to understand how binding interfaces can dynamically change at planar protein interaction surfaces and indicate the complexities that must be incorporated into designing molecules that target them. These results also highlight how consideration of intramolecular stabilizing interactions of the bound molecules can lead to higher K_d_s against these sites, without having to increase the molecular weight of these compounds in the search for new intermolecular interactions. The insights presented here should aid the development of more potent and specific molecules against eIF4E with potential therapeutic applications.

## Materials and Methods

### Peptide Reagents

All peptides were ordered from and synthesized by Mimotopes, Clayton Austrailia. All peptides were HPLC purified to >90% purity.

### Protein Expression and Purification

Full-length human eIF4E was expressed and purified as described previously. [16], [20].

### Biotinylation of eIF4E for Use in Phage Display Panning

Aliquots of eIF4E were kept frozen at 80°C until use. Sulfo-NHS–LC–LC biotin was added in an eqimolar ratio to a solution of eIF4E at a concentration of a 100 µM and incubated at room temperature. After 1 h, unreacted biotin were removed by passing the solution over a fast desalting column (equilibrated with Phosphate Buffered Saline) twice. Biotinylated eIF4E was stored at 4°C for a maximum period of up till 1 week.

### Phage Display

An M13 phage library (Ph.D.-12, New England Biolabs) encoding random 12-mer peptides at the NH_2_ terminus of pIII coat protein (2.7 × 10^9^ sequences) was used. Biotinylated full length eIF4E was loaded onto 10 µl of steptavdin M280 magnetic Dynabeads (Invitrogen). The loaded beads were incubated with blocking buffer (20 mM HEPES pH 7.6, 0.1 M KCL, 0.5% Tween20, 2% BSA) for 1 h at room temperature, washed with buffer W (20 nM HEPES pH 7.6, 0.1 M KCL, 0.5% Tween 20), and incubated in buffer W at room temperature with 4 × 10^10^ phages. Magnetic M280 beads were the washed 8 times in buffer W. Bound phages were eluted with 0.2 M glycine (pH 2.2) and neutralized with 1 M Tris (pH 9.1). The eluted phages were amplified as instructed by the manufacturer. The selection process was repeated for three cycles. Phage plaques from the final round were picked and amplified as described by the manufacturer and sequenced.

**Table 4:** Data collection and refinement statistics.

Unit cell dimensions (Å)	a = 60.66, b = 38.17, c = 121.76, α = γ = β = 90°
Resolution (Å)	2.16
Space group	P2_1_
Temp (K)	100
Collected reflections	282,355
Unique reflections	30,410
R Sym (%)	19.58 (74.52)
I/sigmaI	5.22(1.08)
R factor (%)	23.92
R free (%)	27.86
RMS bonds (Å)	0.0072
RMS angle (°)	1.142
% Completeness	99.3% (94.5%)
Average B value (Å^2^)	
Chain A	13.24
Chain B (peptide)	17.71
Chain C	12.73
Chain D (peptide)	17.94
m^7^GTP (chain E)	13.33
m^7^GTP (chain F)	13.46
Molecules in Asymmetric Unit	
eIF4E	2
Number of solvent molecules	205
Ligands	
m^7^GTP	2
Peptide	2
Ramachandran data:	
Favoured regions (%)	94.40
Additionally allowed regions (%)	5.1
Generously allowed regions (%)	0.60
Disallowed regions (%)	0.00

Crystallographic data and refinement statistics for eIF4E in complex with m^7^GTP and eIF4G1-D5S (PDB ID: 4AZA).

### Flourescence Based Thermal Stability Measurements

A fluorescence based thermal shift assay was used to screen and rank the rationally designed eIF4E binding derivative peptides. The fluorescent dye Sypro Red (Invitrogen) was used to monitor thermal denaturation of eIF4E. Binding of the dye molecule to eIF4E, as it unfolds due to thermal denaturation, results in a sharp increase in the fluorescence intensity. The midpoint of this transition is termed the T_m_. The thermal shift assay was conducted in a LightCycler (Roche). Protein samples studied were made up to a total volume of 50 µl in PBS (Phosphate Buffered Saline) with Sypro Red, (Invitrogen, 5000× DMSO stock) at a 3.125× concentration. The final protein concentration was 10 µM. Protein samples were incubated with derivative peptides at a concentration of 100 µM. The plate was heated from 20 to 90°C with a heating rate of 1°C/min. The fluorescence intensity was measured with Ex/Em:533/640 nm.

The fluorescence data against temperature derived from the LightCycler were fitted to Eq. (1) [21] to obtain ΔH_u_, ΔC_pu_, and T_m_ by nonlinear regression using the program Prism 4.0, Graphpad:
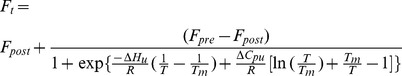
(1)where F_t_ is the fluorescence intensity at temperature T; T_m_ is the midpoint temperature of the protein-unfolding transition, F_pre_ and F_post_ are the pretransitional and posttransitional fluorescence intensities, respectively, R is the gas constant, ΔH_u_ is the enthalpy of protein unfolding, and ΔC_pu_ is the heat capacity change on protein unfolding. In the absence of ligand, T_m_ = T_0_, ΔC_pu_ = ΔC^T0^_pu_, and ΔH_u_ = ΔH^T0^_u_.

To calculate the ligand-binding affinity at T_m_ for the derivative eIF4E binding peptides, Eq. (2) [21]was used:
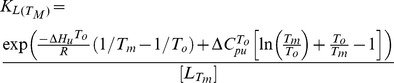
(2)


To compare binding affinities for the derivative peptides to eIF4E calculated from the thermal shift data, the binding affinity at temperature T (K_L(T)_) must be calculated. K_L(T)_ can be calculated from K_L(Tm)_ using Eq. (3): [21]

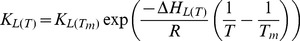
(3)where K_L(T)_ is the ligand association constant at temperature T, and ΔH_L(T)_ is the van’t Hoff enthalpy of binding at temperature T. The value of ΔH_L(T)_ was taken to be -5 kcal/mol [21].

### Surface Plasmon Resonance

For stock peptide solutions, the compounds were dissolved in 100% DMSO to a concentration of 10 mM; further dilutions of the peptide stock solutions into DMSO and/or running buffer were performed immediately prior to analysis. Running buffer consisted of 10 mM Hepes pH 7.6, 0.15 M NaCl, 1 mM DTT and 0.1% Tween20. Stock/DMSO diluted peptide solutions were diluted into 1.03× running buffer to make a peptide solution with 3% DMSO final concentration. Working concentrations of peptide were reached with further dilution of samples into running buffer which contained 3% DMSO. Surface Plasmon resonance experiments were performed on a Biacore T100 machine.

Pure eIF4E was immobilized on a CM5 sensor chip. The CM5 chip was conditioned with a 6 s injection of 100mM HCL, followed by a 6 s injection of 0.1% SDS and completed with a 6 s injection of 50 mM NaOH at a flow rate of 100µl/min. Activation of the sensor chip surface was performed with a mixture of NHS (115 mg ml^−1^) and EDC (750 mg ml^−1^) for 7 min at 10 µl min^−1^. Purified eIF4E was diluted with 10 mM sodium acetate buffer (pH 5.0) to a final concentration of 0.5 µM with m^7^GTP present in a 2∶1 ratio in order to stabilize eIF4E. The amount of eIF4E immobilized on the activated surface was controlled by altering the contact time of the protein solution and was approximately 1000 RU. After the immobilization of the protein, a 7-min injection (at 10 µl min^−1^) of 1 M ethanolamine (pH 8.5) was used to quench excess active succinimide ester groups.

Six buffer blanks were first injected to equilibrate the instrument fully and then a solvent correction curve was performed followed by a further two blank injections. The solvent correction curve was setup by adding varying amount of 100% DMSO to 1.03x running buffer to generate a range of DMSO solutions (3.8%, 3.6%, 3.4%, 3.2%, 3%, 2.85%, 2.7% and 2.5% respectively). Using a flow rate of 50 µl/min, compounds were injected for 60 s and dissociation was monitored for 180 s. The data collection rate was 10 Hz. K_d_s were determined using the BiaEvaluation software (Biacore) and calculated from both the response of the eIF4E coated CM5 chips at equilibrium and also kinetically from the dissociation and association phase data for each of the peptides. Both the equilibrium and kinetic data were fitted to 1∶1 binding models. Each individual peptide K_d_ was determined from three separate titrations. Within each titration at least two concentration points were duplicated to ensure stability and robustness of the chip surface.

### Computer Simulations

ACE and NME caps were added to both eIF4E and the eIF4G1 through the use of t-leap in AMBER11. [22] The MD simulations were performed using the TIP3P water model [23], and a minimum distance of 12A was set between the solute and solvation box boundary. The forcefield ff99SB [24] was chosen for all simulated systems. Each system underwent the following 3-phase minimization protocol: 1) Steepest descent method for 1000 cycles, with the solutes frozen with a force constant of 500 kcal mol^−1^ angstrom^−2^, 2)Steepest descent method for 1000 cycles, with the solvent frozen with a force constant of 500 kcal mol^−1^ angstrom^−2^ and 3)Steepest descent method for 1000 cycles, followed by 1000 cycles of conjugate gradient method for another 1000 cycles. This was done on the whole system. The system was then heated from 1F to 300F over 30 ps. The MD simulations were run using both the SANDER and CUDA module of the AMBER11 package on. A step size of 2fs with the constraint algorithm SHAKE [25] was used. Two replicates with different random seed numbers were carried out for each system, each for a length of 50 ns, for a total of 100 ns per system.

### Crystallization

The eIF4E:eIF4G1-D5S complex was crystallized by vapor diffusion using the sitting drop method. Crystallization drops were setup with eIF4E, eIF4G1-D5S peptide and m^7^GTP at concentrations of 75 µM, 300 µM and 150 µM respectively. Sitting drops were set up in 48 well Intelli-Plates (Hampton research) with 1 µl of the protein sample mixed with 1 µl of the mother-well solution. Crystals grew over a period of one week in 10–20% of Polyethylene glycol monomethyl ether 5,000 and 100 mM Hepes or Bis-Tris at pHs of 6.5, 7.0 and 7.5. For X-ray data collection at 100 K, crystals were transferred to an equivalent mother liquor solution containing 20% (v/v) glycerol and then flash frozen in liquid nitrogen.

### Data Collection and Refinement

The data was collected on a X8 Proteum rotating anode source (Bruker) using a CCD detector. The crystal diffracted to a resolution of 2.2 Å and was integrated and scaled using PROTEUM2 (Bruker). The initial phases of the ternary complexed crystals of eIF4E were solved by molecular replacement with the program PHASER [26] using the human eIF4E structure complexed with the eIF4G1 peptide (PDB accession code: 2W97) as a search model. The starting models were subjected to rigid body refinement and followed by iterative cycles of manual model building in Coot and restrained refinement in Refmac 6.0. [27] m^7^GTP was added into clearly visible electron density. REFMAC library files for the ligand molecule were generated using PRODRG. [28] Models were validated using PROCHECK [29] and the MOLPROBITY webserver. [30] Final models were analysed using PYMOL (Schrödinger). See table 4 for data collection and refinement statistics. The eIF4E complex structure has been deposited in the PDB under the submission code 4AZA.

## Supporting Information

Figure S1 **SPR sensograms of eIF4E immobilized via amine coupling on a CM5 chip with eIF4E interacting peptides.** SPR sensograms showing titrations of the peptides used to study the relevance of individual amino acid changes observed in the phage derived sequence against eIF4E.
(TIF)

Figure S2 **SPR sensograms of eIF4E immobilized via amine coupling on a CM5 chip with eIF4E interacting peptides.** SPR sensograms showing titrations of the peptides used to study the N-capping motif in the eI4G1 wild type sequence.
(TIF)

Figure S3 **SPR sensograms of eIF4E immobilized via amine coupling on a CM5 chip with eIF4E interacting peptides.** SPR sensograms showing titrations of the PHAGESOL and VL-PHAGESOL peptides used to study the relationship of amino acid positions 8 and 12 in relation to the presence of the amino acid V present at position 10
(TIF)

Figure S4 **Distribution plots of the relative positions of the helical portions of the eIF4E bound peptides with respect to the surface of the eIF4E. A)** Plots showing the distribution of the relative positions of the helical portions of the eIF4E bound peptides with respect to the surface of the protein throughout their individual simulations for the PHAGESOL and VL-PHAGESOL variant peptides. The plots show the distinct conformational differences of the peptides in their interactions with eIF4E. **B)** Schematic demonstrating how the relative position of the eIF4E interacting peptide was calculated in relation to the binding site. The relative position of the helix was derived by defining it as the centre of mass of residues 8 to 12 of the peptide. Two distance measurements were made from this point (A and B) to two respective points on the surface of eIF4E, which were defined as the centre of mass of residues V69 to W74 (indicated with yellow on protein surface) and residues W130–135 (indicated with blue on the protein surface). The distance between these two points were also measured (C). These measurements were then used to calculate the perpendicular distance from the peptide to point A on the line defined by A and B and plotted on the X axis. On the Y axis the height of the perpendicular drop from the peptide to the surface of eIF4E as defined by the line running from points A to B was plotted. These calculations were applied to all frames from the simulations.
(TIF)
