# Supplementary figures and images for: Improved eIF4E Binding Peptides by Phage Display Guided Design: Plasticity of Interacting Surfaces Yield Collective Effects
Source: PLoS One. 2012 Oct 19;7(10):e47235. doi: 10.1371/journal.pone.0047235 (PMC3477164; doi:10.1371/journal.pone.0047235)

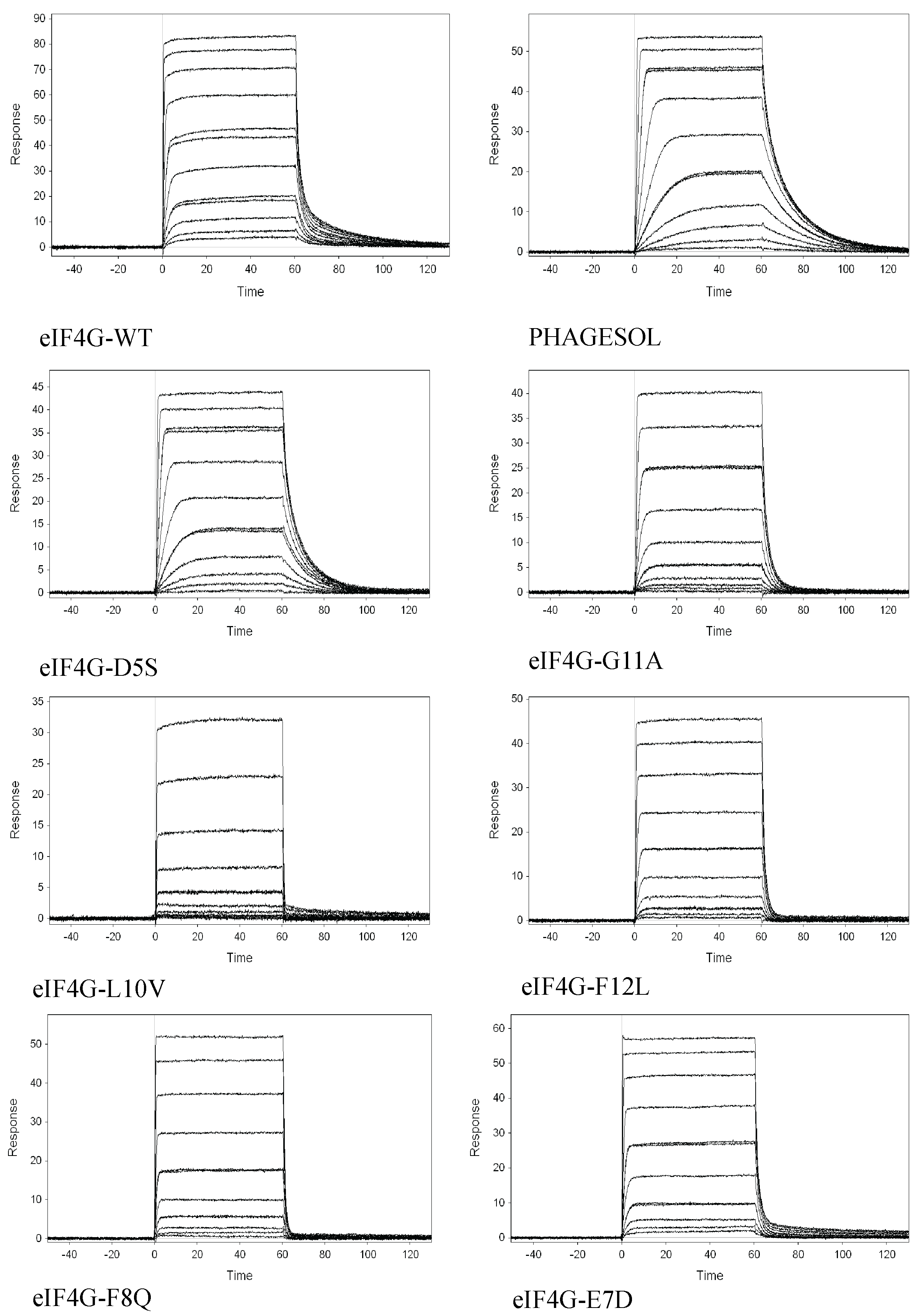

Supplement: Figure S1 — SPR sensograms of eIF4E immobilized via amine coupling on a CM5 chip with eIF4E interacting peptides. SPR sensograms showing titrations of the peptides used to study the relevance of individual amino acid changes observed in the phage derived sequence against eIF4E. (TIF) [file pone.0047235.s001.tif]

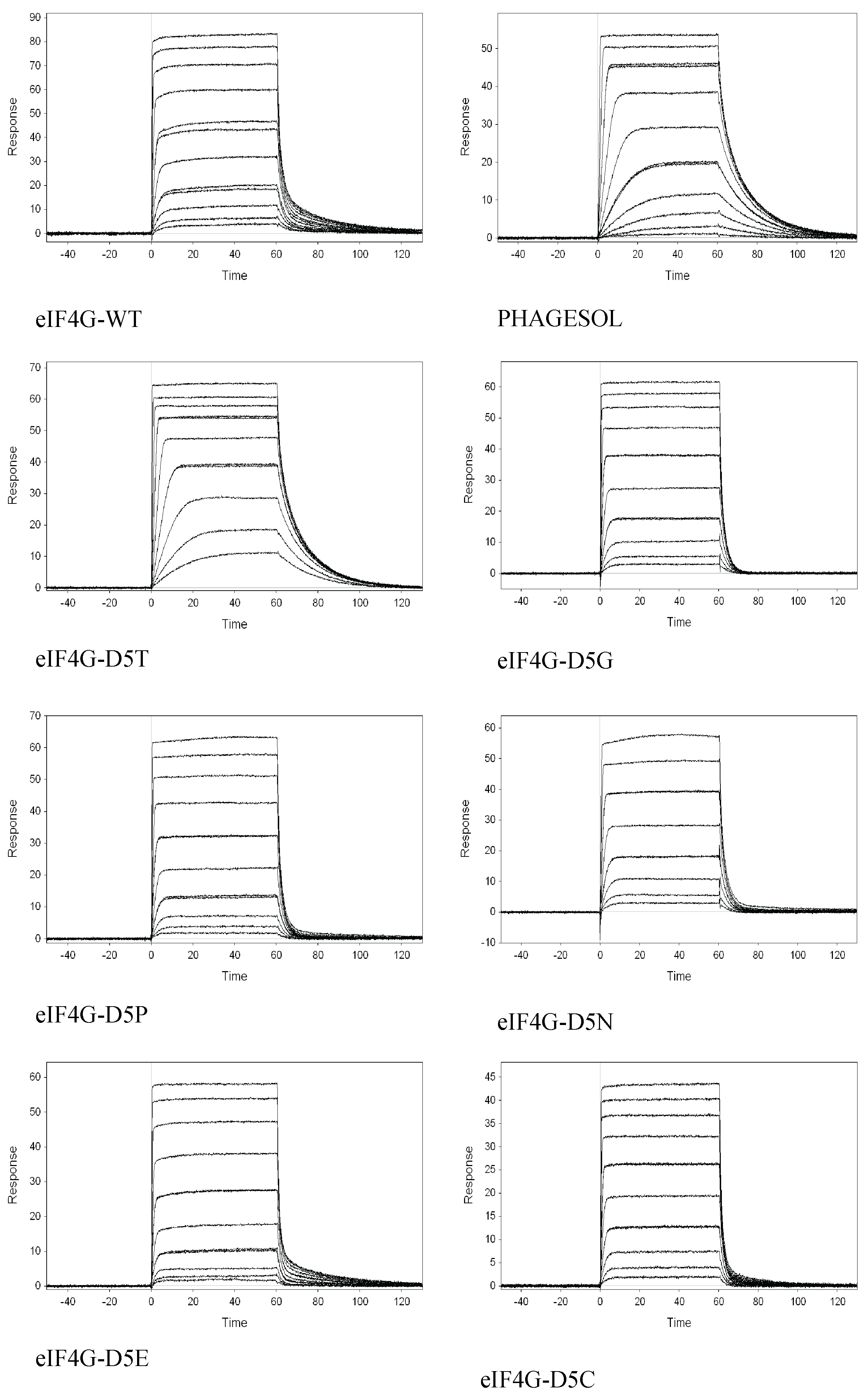

Supplement: Figure S2 — SPR sensograms of eIF4E immobilized via amine coupling on a CM5 chip with eIF4E interacting peptides. SPR sensograms showing titrations of the peptides used to study the N-capping motif in the eI4G1 wild type sequence. (TIF) [file pone.0047235.s002.tif]

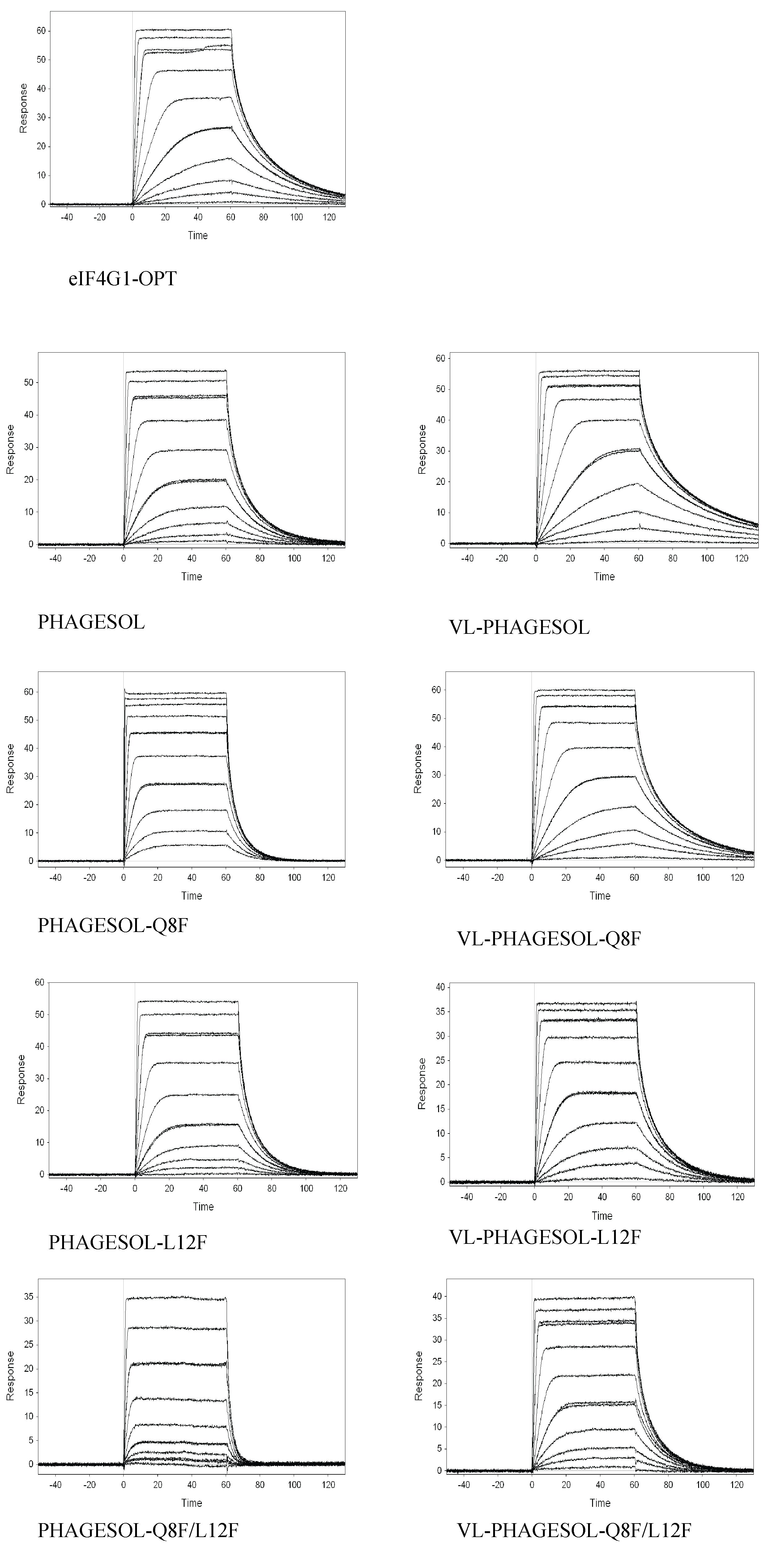

Supplement: Figure S3 — SPR sensograms of eIF4E immobilized via amine coupling on a CM5 chip with eIF4E interacting peptides. SPR sensograms showing titrations of the PHAGESOL and VL-PHAGESOL peptides used to study the relationship of amino acid positions 8 and 12 in relation to the presence of the amino acid V present at position 10 (TIF) [file pone.0047235.s003.tif]

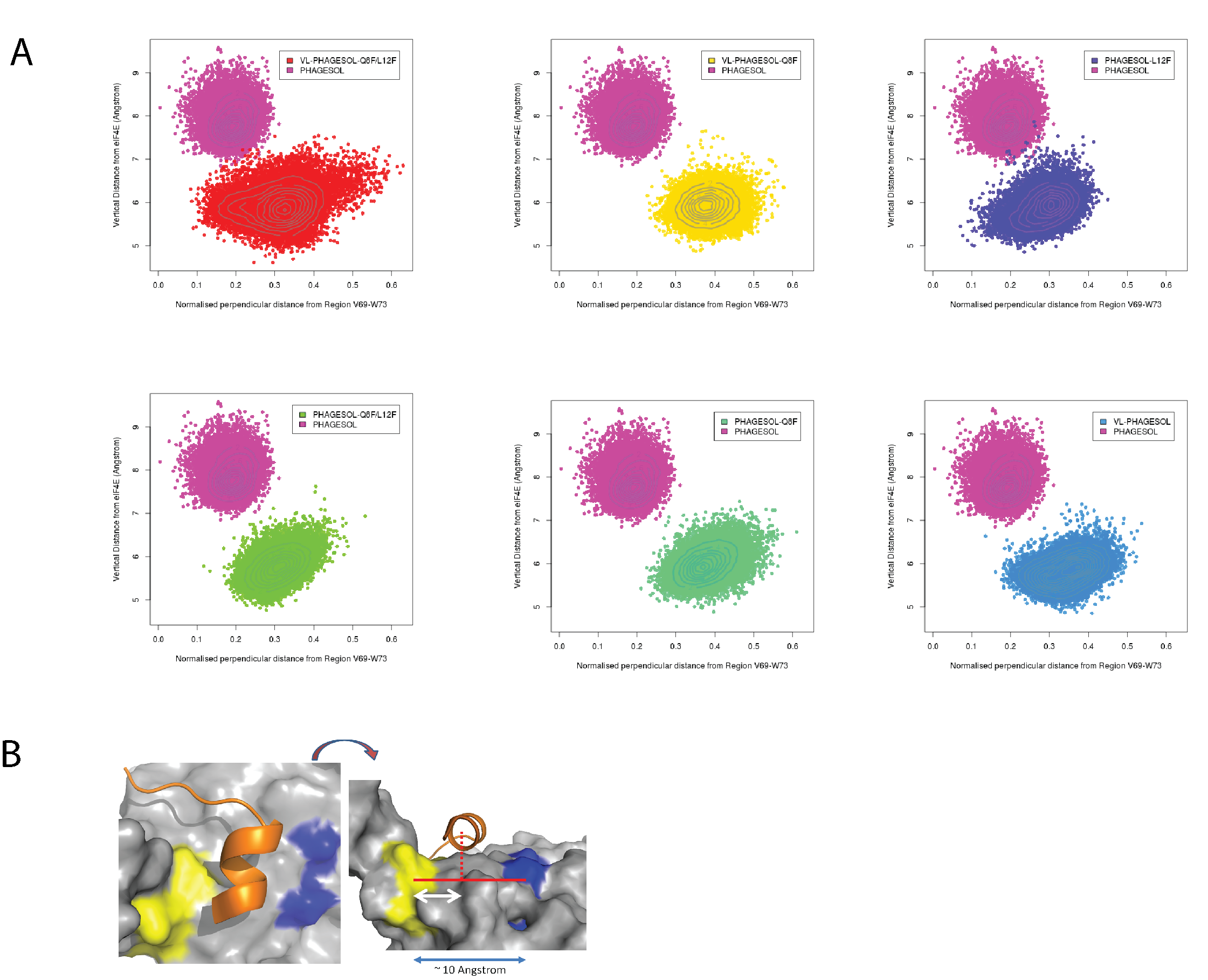

Supplement: Figure S4 — Distribution plots of the relative positions of the helical portions of the eIF4E bound peptides with respect to the surface of the eIF4E. A) Plots showing the distribution of the relative positions of the helical portions of the eIF4E bound peptides with respect to the surface of the protein throughout their individual simulations for the PHAGESOL and VL-PHAGESOL variant peptides. The plots show the distinct conformational differences of the peptides in their interactions with eIF4E. B) Schematic demonstrating how the relative position of the eIF4E interacting peptide was calculated in relation to the binding site. The relative position of the helix was derived by defining it as the centre of mass of residues 8 to 12 of the peptide. Two distance measurements were made from this point (A and B) to two respective points on the surface of eIF4E, which were defined as the centre of mass of residues V69 to W74 (indicated with yellow on protein surface) and residues W130–135 (indicated with blue on the protein surface). The distance between these two points were also measured (C). These measurements were then used to calculate the perpendicular distance from the peptide to point A on the line defined by A and B and plotted on the X axis. On the Y axis the height of the perpendicular drop from the peptide to the surface of eIF4E as defined by the line running from points A to B was plotted. These calculations were applied to all frames from the simulations. (TIF) [file pone.0047235.s004.tif]
